# DNA polymerase θ-mediated repair of high LET radiation-induced complex DNA double-strand breaks

**DOI:** 10.1093/nar/gkad076

**Published:** 2023-02-20

**Authors:** Geunil Yi, Yubin Sung, Chanwoo Kim, Jae Sun Ra, Hirokazu Hirakawa, Takamitsu A Kato, Akira Fujimori, Hajin Kim, Kei-ichi Takata

**Affiliations:** Center for Genomic Integrity, Institute for Basic Science, Ulsan 44919, Republic of Korea; Department of Biological Sciences, Ulsan National Institute of Science and Technology, Ulsan 44919, Republic of Korea; Center for Genomic Integrity, Institute for Basic Science, Ulsan 44919, Republic of Korea; Department of Biomedical Engineering, Ulsan National Institute of Science and Technology, Ulsan 44919, Republic of Korea; Center for Genomic Integrity, Institute for Basic Science, Ulsan 44919, Republic of Korea; Department of Charged Particle Therapy Research, Institute for Quantum Medical Science, Chiba 263-8555, Japan; Department of Environmental & Radiological Health Sciences, Colorado State University, Colorado 80523, USA; Department of Charged Particle Therapy Research, Institute for Quantum Medical Science, Chiba 263-8555, Japan; Center for Genomic Integrity, Institute for Basic Science, Ulsan 44919, Republic of Korea; Department of Biomedical Engineering, Ulsan National Institute of Science and Technology, Ulsan 44919, Republic of Korea; Center for Genomic Integrity, Institute for Basic Science, Ulsan 44919, Republic of Korea; Department of Biological Sciences, Ulsan National Institute of Science and Technology, Ulsan 44919, Republic of Korea

## Abstract

DNA polymerase θ (POLQ) is a unique DNA polymerase that is able to perform microhomology-mediated end-joining as well as translesion synthesis (TLS) across an abasic (AP) site and thymine glycol (Tg). However, the biological significance of the TLS activity is currently unknown. Herein we provide evidence that the TLS activity of POLQ plays a critical role in repairing complex DNA double-strand breaks (DSBs) induced by high linear energy transfer (LET) radiation. Radiotherapy with high LET radiation such as carbon ions leads to more deleterious biological effects than corresponding doses of low LET radiation such as X-rays. High LET-induced DSBs are considered to be complex, carrying additional DNA damage such as AP site and Tg in close proximity to the DSB sites. However, it is not clearly understood how complex DSBs are processed in mammalian cells. We demonstrated that genetic disruption of *POLQ* results in an increase of chromatid breaks and enhanced cellular sensitivity following treatment with high LET radiation. At the biochemical level, POLQ was able to bypass an AP site and Tg during end-joining and was able to anneal two single-stranded DNA tails when DNA lesions were located outside the microhomology. This study offers evidence that POLQ is directly involved in the repair of complex DSBs.

## INTRODUCTION

Ionizing radiation (IR) therapy is frequently used in the treatment of cancer and is believed to exert its effects by DNA breaks in cells. Radiation from heavy charged particles such as carbon ions produced by particle accelerators has a high linear energy transfer (LET) and releases most of its energy within a short range, called the Bragg peak. If the Bragg peak is focused on the tumor, damage to surrounding normal tissues can be minimized compared to the more commonly used low LET radiation such as gamma- or X-rays (1,2). A spread-out Bragg peak (SOBP) beam, which consists of various monoenergetic Bragg peak beams to extend the Bragg peak region to precisely cover the tumor area, has been demonstrated to be effective in radiation therapy (3). DNA double-strand breaks (DSBs) generated by high LET heavy ion radiation (hiLET-DSBs) are more complex than low LET-induced DSBs (loLET-DSBs), as they carry additional DNA damage such as apurinic/apyrimidinic (AP) site and thymine glycol (Tg) in close proximity to the DSB sites, making it more cytotoxic per unit dose than low LET radiation (4,5). X-rays and carbon ions can generate both clean and complex DSBs. However, carbon ions produce more complex DSBs, and X-rays produce relatively ‘clean’ DSBs (6).

There are three major pathways for the repair of DSBs. Broken DNA ends are joined via non-homologous end joining (NHEJ) and DNA polymerase theta-mediated end joining (TMEJ) (7) or repaired by homologous recombination (HR) if a homologous sister chromatid is available as a template for accurate repair (8). It has been reported that NHEJ is ineffective at joining complex DSBs, but HR can contribute to the repair of complex DSBs after removing DSB-proximal DNA damage by DNA end resection (9–11). However, whether TMEJ is involved in the process is currently unknown.

Complex DSBs are more mutagenic and cytotoxic than ‘clean’ breaks (6,12,13). The inefficient repair of complex DSBs likely reflects the inhibition of NHEJ by additional damage, resulting in an obstruction of the ligation step by base damage and AP sites (14,15). KU binding on complex DSB ends may also be prevented by base damage as well as base excision repair (BER) or single-strand break (SSB) repair proteins bound to complex DSBs (16). BER enzymes binding damaged bases may cleave the joined DNA products resulting in secondary DNA break formation (17). Since base damage inhibits end-joining, the majority of complex DSBs are likely to be repaired after removing DSB-proximal DNA damage. DNA end resection can clean up DSB-proximal DNA damage to promote the repair of complex DSBs (18–21). The dRP/AP lyase of KU can also excise AP sites near broken ends (14). However, it has not been fully investigated how these resected complex DSB ends are processed in mammalian cells. We hypothesized that DNA polymerase θ (POLQ) is an important factor to repair complex DSBs because POLQ is able to perform end-joining of resected DSB ends (22–25) and to bypass AP sites and Tg lesions during DNA synthesis (26,27).

POLQ is a major factor in controlling radiosensitivity (24,28). It participates in DSB end-joining through microhomology-mediated end-joining (MMEJ), specifically by a process designated as TMEJ (29). MMEJ occurs in yeast, which does not encode a *POLQ* gene (30). MMEJ can be performed by POLQ and also by factors involved in NHEJ, although the mechanisms are different. NHEJ can ligate blunt ends or compatible ends with 1–4 ssDNA residues, while POLQ can process longer resected DNA ends utilizing a microhomology of 2–6 bp (7). TMEJ is a preferred term to refer to the POLQ activity (31). TMEJ introduces short DNA deletions with overlapping microhomology while protecting against catastrophic large deletions (32,33). The requirement of POLQ for the viability of HR-deficient cells underscores the importance of POLQ-dependent genome protection function including TMEJ and post-replicative ssDNA gap sealing (34–37). POLQ is a unique enzyme composed of an N-terminal helicase-like domain (HLD), a central domain, and a C-terminal A-family DNA polymerase domain (38). The HLD and polymerase domains both contribute to TMEJ (33,39,40). The HLD retains ATPase activity but no DNA unwinding activity (38,41). It can disassemble RPA from ssDNA and can bridge two DNA molecules mimicking resection intermediates (42). POLQ is an error-prone polymerase with the ability to perform translesion synthesis (TLS) across lesions such as AP site and Tg (26,27). The C-terminal POLQ polymerase domain known as POLQ (QM1) retains the activity to perform TLS (23), to anneal broken ends utilizing microhomology (25), and to extend DNA from highly mismatched primer ends (22,23,43).

Here, we provide evidence for the specific role of POLQ in promoting the end-joining of complex DSBs. Genetic disruption of *POLQ* in human cells led to an increase in the hallmarks of genomic instability and cellular hypersensitivity following SOBP carbon ion irradiation. Using biochemical and single-molecule FRET experiments, we found that POLQ efficiently anneals and extends substrates mimicking complex DSBs. Our findings provide new insights into the mechanisms of how hiLET-DSB is repaired in mammalian cells and suggest that the inhibition of POLQ may augment the efficacy of heavy ion radiation therapy.

## MATERIALS AND METHODS

### shRNA vectors

shRNA vectors were purchased from Horizon: shPRKDC (DNA-PKcs) (V2LHS_94774) and shControl (RHS4346).

### siRNA transfection

The FANCD2-specific Stealth RNAs (designated ‘siFD2’, 5’-AAUGAACGCUCUUUAGCAGACAUGG-3’) and ON-TARGETplus Non-Targeting siRNAs as a negative control designated ‘siC’ (Thermo scientific) were purchased from Invitrogen and Thermo Scientific, respectively. The siRNAs were introduced into wild type or *POLQ* knockout U2OS cells. Twenty-four hours prior to transfection, cells were plated in a 6-well plate at 2.0 × 10^5^ cells/well. For each well, 5 pmol of siRNAs was diluted into 250 μl of Opti-MEM (Invitrogen). In a separate tube, 5 μl of Lipofectamine RNAiMAX reagent (Invitrogen) was diluted into 250 μl of Opti-MEM and incubated at room temperature for 10 min. The Lipofectamine RNAiMAX dilution was added into the diluted siRNA duplex and incubated at room temperature for 20 min. Before the transfection, medium was replaced with fresh 2.5 ml of DMEM supplemented with 10% fetal bovine serum for each well. The Lipofectamine RNAiMAX-siRNA complex was added dropwise to the cells and incubated at 37°C. After 24 h the cells were washed, trypsinized, and plated with fresh DMEM medium supplemented with 10% fetal bovine serum and 1% penicillin-streptomycin (Invitrogen). To measure the levels of proteins, whole cell crude extracts were prepared 48 h after the RNA transfection and analyzed by immunoblotting.

### Irradiation

Particle-based irradiation experiments were carried out at the National Institutes for Quantum Science and Technology (QST) in Chiba, Japan. Carbon ions were accelerated to 290 MeV/nucleon using the Heavy Ion Medical Accelerator in Chiba (HIMAC) and spread out with a ridge filter for 60 mm width of spread-out Bragg peak (SOBP) at 108.51 mmH_2_O. The monolayer cell culture was irradiated at the center (50 keV/μm of average LET) within the SOBP at a distance of 119 mm from the entrance. X-ray irradiation was performed using RS-2000 Biological Irradiator (Rad Source Technologies) or Pantac HF-320S (Shimadzu).

### ATPlite survival experiments

For the ATPlite assay (PerkinElmer), 1250 cells per well were plated into white 96-well plates and incubated 24 h prior to inducing DNA damage. The cells were then incubated with DNA damage-inducing agents for the indicated time. After incubation, the cells were immediately lysed and assayed for ATPlite luminescence as described in the manufacturer's instructions.

### Clonogenic survival assay

Cells were plated at a density of 1.0 × 10^6^ cells/T-75 flask or 1.0 × 10^5^ cells/ 60 mm plate incubated for 24 h prior to DNA damage induction. Groups of plates were exposed to indicated doses of DNA damage-inducing agents for 1 h, X-rays, or carbon ions. They were harvested with trypsin/EDTA and plated again at a density of 50–50 000 cells/100 mm dish depending on the dose of agents or irradiation. After 11–14 days of incubation at 37°C, plates were fixed and stained with absolute methanol and 1% methylene blue in 70% ethanol. Colonies which consist of >30 cells were counted and scored.

### Antibodies

We used the following antibodies: A300-516A (Thermo Fisher), polyclonal anti-DNA-PKcs 1:5000; EPR2302 (GeneTex, Inc), monoclonal anti-FANCD2 1:2000; T5168 (Merck), monoclonal anti-αTubulin 1:8000; sc73614 (Santa Cruz Biotechnology), monoclonal anti-Vinculin 1:1000; A0545 (Merk), HRP (horseradish peroxidase)-conjugated anti-rabbit IgG 1:10 000; A0168 (Merk), HRP-conjugated anti-mouse IgG 1:10 000.

### Micronuclei assay

U2OS cells were plated at a density of 1 × 10^5^ cells/ well in 4-well chamber slides and irradiated on the following day with 2 Gy of X-rays or carbon ions. The cells were then incubated in the presence of 2 μg/ml cytochalasin-B (Cyt-B) for 48 h at 37 °C, fixed with absolute methanol for 10 s, washed three times by PBS, and dried. The fixed samples were stained with DAPI and mounted using Vectashield Antifade Mounting Medium (Vector Laboratories). Three independent experiments were carried out. In one experiment at least 50 binucleated cells were scored to calculate the number of micronuclei per cell and the percentage of micronuclei-containing cells for each sample. At least 200 binucleated cells were analyzed for each sample.

### Chromosome assay

U2OS cells were plated at a density of 1.5 × 10^6^ cells/100 mm dish a day before irradiation. The cells irradiated by X-rays or carbon ions were cultured with 100 ng/ml of Colcemid (Gibco) for 3 h at 37 °C to arrest at metaphase. They were harvested with trypsin/EDTA and suspended in 600 μl of pre-warmed 75 mM of potassium chloride at 37 °C for 15 min. After fixation with a Carnoy's solution (3:1 methanol to acetic acid), the cells were dropped onto glass slides, allowed to dry, and stained with 5% Giemsa in Gurr's buffer. Chromosomal aberration analysis was carried out under Olympus BX53 microscope. At least 33 metaphase spread images were analyzed for each sample in one experiment.

### Microhomology-mediated end-joining assay

POLQ (QM1), a truncated version of human POLQ enzyme consisting of residues 1792–2590 was purified as described (44). Klenow Fragment (KF) (exo^−^) was purchased from Promega. POLQ (QM1) was diluted in buffer containing 37.5 mM Tris–HCl pH 8.0, 40 mM NaCl, 2.5 mM DTT, 0.125% BSA, 0.0125% Triton X-100 and 6.25% glycerol. KF (exo^−^) was diluted in buffer containing 50 mM Tris–HCl pH 7.5, 1 mM DTT, 0.1 mM EDTA and 50% glycerol. POLQ (QM1) reaction mixtures (10 μl) contained 30 mM Tris–HCl pH 8.0, 6.4 mM NaCl, 0.4 mM DTT, 0.02% BSA, 0.01% Triton X-100, 0.5% glycerol, 5 μM of each dNTP and 100 nM of the primer-template (Supplementary Table S1). KF (exo^−^) reaction mixtures (10 μl) contained 50 mM Tris–HCl pH 7.2, 10 mM MgSO_4_, 0.1 mM DTT, 5 μM of each dNTP and 100 nM of the primer-template (Supplementary Table S1). After incubation at 37°C for 20 min, reactions were terminated by adding 10 μl of formamide stop buffer (98% deionized formamide, 10 mM EDTA pH 8.0, 0.025% Xylene cyanol FF, 0.025% Bromophenol blue) and boiling at 95 °C for 3 min. Products were electrophoresed on a denaturing 20% polyacrylamide–7 M urea gel and analyzed with an Amersham Typoon Biomolecular Imager. For analysis, N_1_ refers to the product with only one nucleotide incorporated and N_0_ refers the primer. The total product was defined as the band density ≥N_1_ divided by the intensity of ≥N_0_. The ≥ Full product was defined as the band density ≥ fully extended product divided by the intensity of N_0_. The bypass product at position N was defined as the band density ≥N + 1 divided by the intensity of ≥N_0_. The bypass efficiency was defined as [bypass product]/[total product].

### Preparation of DNA substrates

Tetrahydrofuran (THF)-containing oligonucleotides were synthesized with biotin and amino modifications, and purified by HPLC (Integrated DNA Technologies, USA). A thymine glycol (Tg) containing oligonucleotides were synthesized with biotin and amino modifications (Bioneer, South Korea), and subsequently purified with PAGE. The oligonucleotides were fluorescently labeled at the amine groups by standard assays using Cy3 or Cy5 NHS esters (Lumiprobe, USA) for smFRET measurements (Supplementary Table S1).

### Single-molecule FRET measurements

Single-molecule fluorescence signals were detected using a custom-built total internal reflection fluorescence microscope. The surface was coated with NeutrAvidin (ThermoFisher Scientific, USA) and DNA substrates were immobilized via the biotin–NeutrAvidin interaction. Excess unbound DNA was washed away, and imaging buffer containing 30 mM Tris–HCl pH 8.0, 6.4 mM NaCl, 0.4 mM DTT, 0.02% BSA, 0.01% Triton X-100, 0.5% glycerol, 1 mg/ml glucose oxidase (from Aspergillus niger VII; Sigma, USA), 0.04 mg/ml catalase (from bovine liver; Sigma, USA), and 3 mM Trolox was added, followed by 40 nM POLQ (QM1) or 1 nM KF (exo^−^) and 40 nM Cy5 labeled oligos to anneal two oligos with microhomology. 5 μM dATP (ThermoFisher Scientific, USA; R0181) was added to polymerize on the annealed substrate. Fluorescence movies were recorded using an EMCCD camera (iXon Ultra 897; Oxford Instruments, UK) at the rate of 100 ms per frame. All measurements were repeated more than three times.

### Single-molecule data analysis

Traces containing only a single pair of Cy3 and Cy5 were selected by examining signal intensities and photobleaching steps. Apparent FRET efficiency was calculated as *E*_FRET_ = (*I*_A_− *βI*_D_)/(*I*_D_ + *I*_A_), where *I*_D_ and *I*_A_ are the intensities of the donor and acceptor dyes, respectively, after correcting for the direct excitation of the acceptor by the green laser by subtracting the average acceptor intensity after donor photobleaching. *β* = 0.09 is the correction factor for the donor fluorescence bleeding to the acceptor channel, found by setting the FRET peak of donor-only population at zero. Each FRET histogram was built from more than 30 movies that contained more than 5000 traces. Each FRET value in a histogram was represented by *E*_FRET_ averaged over 25 frames. For dwell time analysis, sHaRPer (serialized Handshaking Repeated Permutation with end removal) method was used to obtain dwell times longer than the duration of a single trace. In each condition, more than 5000 traces were binarized with a cut-off FRET value of 0.4. A concatenated trace was created by repeated permutation of the binarized traces for 10 000 without making discontinuity at the junctions. The means and standard errors of the high FRET or low FRET dwell time were obtained from the average dwell times from three concatenated traces.

## RESULTS

### Deletion of POLQ sensitizes human cells to high LET radiation

To study the role of POLQ in the repair of complex DSBs induced by high LET radiation (hiLET-DSBs), we used *POLQ*^−/−^ U2OS cell lines that we previously generated (45). The three independent *POLQ*^−/−^ U2OS clones were similarly hypersensitive to DSB-inducing agents bleomycin, Zeocin, and X-ray irradiation, but not to mitomycin C, temozolomide or hydroxyurea (Supplementary Figure S1). We also confirmed that TMEJ and NHEJ independently contributed to the repair of DSBs; the knockdown of DNA-PKcs in *POLQ*^−/−^ cells led to an increase in sensitivity to bleomycin (Supplementary Figure S2). Lastly, the knockdown of FANCD2 but not POLQ led to an increase in sensitivity to treatment with the ICL-forming agent MMC (Supplementary Figure S3). These results are consistent with previously reported characteristics of *POLQ*-deficient cells (24,28,46).

**Figure 1.**
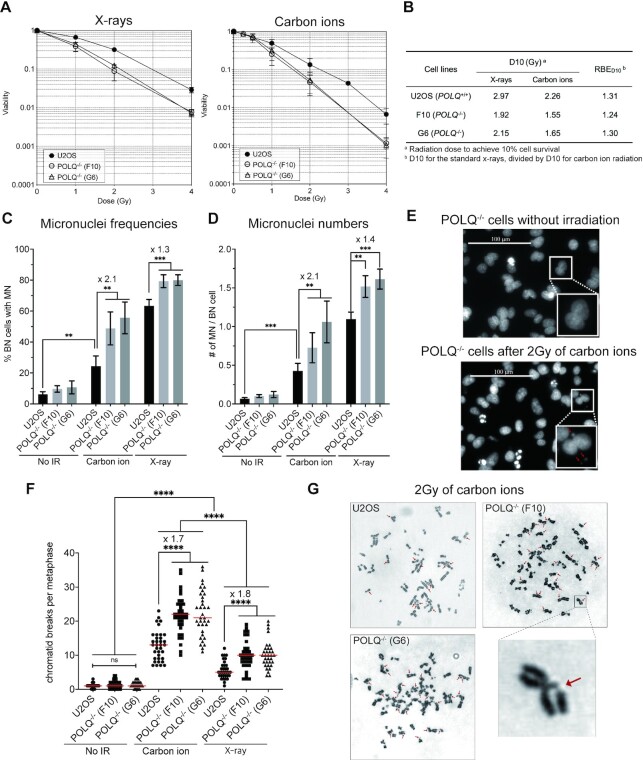
*POLQ* deletion increases cellular sensitivity and aberrant chromosomes after high LET irradiation. (**A**) Effect of *POLQ* deletion on cell survival fraction after X-ray or carbon ion irradiation. *POLQ*-proficient and deficient U2OS cells were irradiated with X-rays or carbon ions. Cells were immediately trypsinized and plated on 100 mm dishes followed by 7–14 days of incubation. After fixation, colonies were stained with methylene blue and counted. All experiments were repeated at least three times and subjected to statistical analysis for each cell line. *Error bars*, standard deviation (S.D). (**B**) Relative biological effectiveness (RBE) values were determined from (A). U2OS cell lines were irradiated with 2 Gy of carbon ions or X-rays and percentages of binucleated cells carrying micronuclei (**C**) and numbers of micronuclei per each binucleated cell (**D**) were measured. At least 200 binucleated cells were analyzed for each sample with three technical replicates. At least 50 binucleated cells were scored for each sample in one experiment. The mean values from three technical replicates were calculated and plotted with error bars. (**E**) Representative images of micronuclei are shown. Arrows point to micronuclei. The unpaired *t*-test for two samples was used to determine the significance. *Columns*, mean; *error bars*, S.D.; *P < 0.1; **P < 0.01; ***P < 0.001. (**F**) The number of chromatid breaks per metaphase in U2OS cell lines irradiated with or without 2 Gy of carbon ions or X-rays was counted. At least 33 metaphase spread images were analyzed for each sample in one experiment. Two *POLQ*^−/−^ cell lines served as biological replicates. The mean values shown as red lines were calculated from independent metaphase images analyzed blindly for each sample. (**G**) Representative images of chromosome spread are shown. Arrows point to chromatid breakages. The unpaired *t*-test for two samples was used to determine the significance. ****P < 0.0001.

If POLQ is involved in joining complex DSBs, *POLQ* deletion should sensitize human cells to high LET radiation. And high LET radiation may sensitize *POLQ*^−/−^ cells more than low LET radiation. We addressed these questions by irradiating human cell lines proficient and deficient for TMEJ with X-rays (low LET) or SOBP carbon ions (high LET). *POLQ*^−/−^ cells were more sensitive to X-rays and carbon ions compared to wild type cells. All tested *POLQ*^−/−^ clones showed a lower level of survival with carbon ions than with X-rays (Figure 1A). In other words, the carbon ion beam required less physical dose than the X-rays to have the same biological effect. This effect was quantified by determining the relative biological effectiveness (RBE) values from Figure 1A as described previously (47). The RBE_D10_ was defined as the ratio of dose for the standard X-rays, divided by the dose for carbon ion radiation required to achieve 10% cell survival (D10_X-ray_/ D10_carbon ion_). The RBE_D10_ for U2OS was calculated as 2.97/2.26 = 1.31. The RBE_D10_ for *POLQ*^−/−^ cell lines F10 and G6 were calculated as 1.92/1.55 = 1.24 and 2.15/1.65 = 1.30, respectively (Figure 1B). All RBE_D10_ values were >1.0, indicating that carbon ions were more toxic than X-rays for all characterized cell lines. It has been reported that RBE_D10_ values of carbon ions to γ-rays (low LET) are significantly >1.00 in the wild type and HR-deficient mammalian cells but not in the NHEJ-deficient cells (48) indicating hiLET-DSBs are hardly processed by NHEJ but repaired by HR following DNA end resection. In our study the RBE_D10_ values were greater than 1.00 in *POLQ*^−/−^ cells, indicating that the response of TMEJ to hiLET-DSBs is different from NHEJ and that TMEJ is one of the DNA end resection-mediated pathways to repair hiLET-DSBs.

### POLQ deletion enhances chromosomal instability more after high LET radiation than after low LET radiation

Since *POLQ*^−/−^ human cells were hypersensitive to carbon ion radiation, we examined whether *POLQ* deletion increases radiation-induced micronuclei, the byproduct of unrepaired DSBs (49) (Supplementary Table S2). The increase in micronuclei formation between *POLQ*-proficient and deficient cells was higher after carbon ion irradiation than X-ray irradiation in terms of both frequency (2.1-fold versus 1.3-fold increase) and number (2.1-fold versus 1.4-fold increase) (Figure 1C,D,E). Increased micronuclei formation after X-ray irradiation in *POLQ*^−/−^ cells is consistent with previously published results (24,28,50). In our experimental condition, X-ray radiation resulted in a higher frequency of micronuclei formation compared to the same dose of carbon ion radiation, which may be because treatment with carbon ions resulted in increased cell death due to more severe damage. Indeed, the morphological hallmarks of apoptosis and mitotic catastrophe (51) were more prominent in cells treated with carbon ions (Supplementary Figure S4).

These radiation-induced micronuclei originated from chromatid breaks. The majority of metaphase spreads displayed chromatid breaks after X-ray or carbon ion irradiation more frequently in *POLQ*^−/−^ cells. The numbers of chromatid breaks detected in metaphase spreads were significantly higher after carbon ion irradiation than after X-ray irradiation (Figure 1F, G). This result is consistent with the significantly higher number of apoptotic cells detected after carbon ion irradiation (Supplementary Figure S4). Carbon ion radiation-induced chromatid breaks were significantly increased in *POLQ*^−/−^ cells than in *POLQ*^+/+^ cells, averaging 12.8, 21.7 and 22.3 in *POLQ*^+/+^, F10 (*POLQ*^−/−^) and G6 (*POLQ*^−/−^) cells, respectively (Supplementary Table S3). These results indicate that POLQ activity is important to reduce DNA breaks after high LET irradiation. The number of chromatid breaks similarly increased 1.8- and 1.7-fold in *POLQ*^−/−^ cells compared to *POLQ^+/+^* cells after low and high LET radiation, respectively, indicating that POLQ can process both loLET-DSBs and hiLET-DSBs.

### POLQ is able to perform TLS across an AP site and Tg in TMEJ

**Figure 2.**
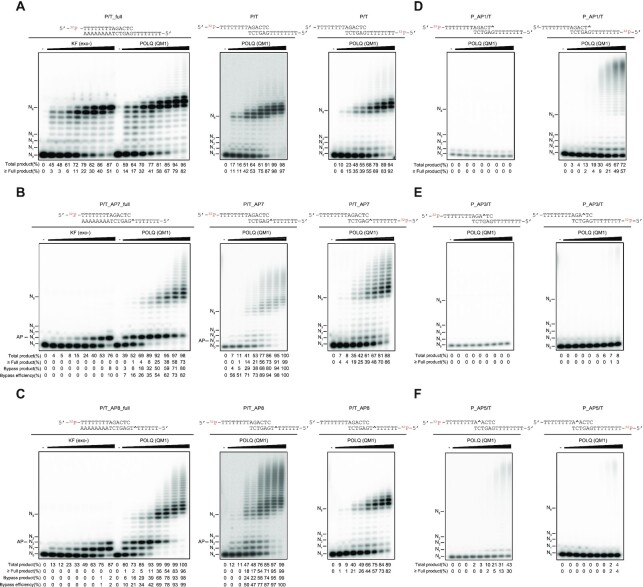
POLQ (QM1) efficiently bypasses an AP site on TMEJ substrates. Increasing concentrations of POLQ (QM1) (0, 0.3125, 0.625, 1.25, 2.5, 5, 10, 20, 40 nM) or KF (exo-) (0, 3.9, 7.8, 15.6, 31.2, 62.5, 125, 250, 500 fM) were incubated with primer-template fully annealed substrates or T-tailed TMEJ substrates carrying no DNA damage (**A**) or an AP site at different positions denoted as ^ (**B–F**). Expected substrates after synapsis formation and the strands labeled with ^32^P are shown above each gel image (A–F). All reaction mixtures had 100 nM substrate and 5 μM dATP, and were incubated at 37°C for 20 min. Locations of unreacted end-labeled primer (N_0_), template base position (N_1_, N_2_, N_3_), full-length product (N_8_), and positions of THF on template (AP) are shown in each gel. The percentage (%) of total product was calculated as [≥N_1_]/[≥N_0_]. The percentage of fully and further extended product (≥ Full) was calculated as [≥N_8_]/[≥N_0_]. The percentage of bypass product for DNA damage at position N was defined as [≥N + 1]/[≥N_0_]. The bypass efficiency was calculated as [the percentage of bypass product]/[the percentage of total product]. These percentages are shown below each lane. Original gels are presented in Supplementary Figure S10.

POLQ (QM1), a truncated version of the human POLQ enzyme consisting of residues 1792–2590 (44) retains polymerase, TMEJ, and TLS activities (22–24,44). Consistent with previously reported results, POLQ (QM1) was able to extend the 3′ primer end of a TMEJ substrate (P/T) (Supplementary Table S1). In contrast, another A-family DNA polymerase Klenow Fragment (KF) (exo-) was able to extend the primer of the fully annealed substrate (P/T_full), but not of the TMEJ substrate (P/T) (Supplementary Figure S5). The TMEJ substrate was designed to undergo synapsis via 6 bps of central microhomology (5’-AGACTC in P and 5’ GAGTCT in T), with the remaining oligos as 5’ overhangs in the presence of POLQ (QM1). Polymerase activity by POLQ (QM1) on this substrate was monitored by extension of the radioactively labeled P or T strand with dATP. POLQ (QM1) similarly extended DNA from the primer (P) and the template (T) (Figure 2A). The P or T strands alone were not extended by POLQ (QM1) (Supplementary Figure S8). This was important to test because some types of oligos can be self-annealed and extended by POLQ (QM1) (23,24), making it difficult to analyze the data. The activity of POLQ to bypass AP sites was previously reported (26,27). POLQ (QM1) was able to bypass the AP lesion on primer-template fully annealed substrates while KF (exo-) was not (Figure 2B, C), although both enzymes showed similar activity on a non-damaged fully annealed substrate (Figure 2A).

To test the activity of POLQ (QM1) in bypassing an AP site on TMEJ substrates, the P/T substrate was used as a control and an AP site was introduced at different locations. We found that POLQ (QM1) is able to bypass an AP site efficiently when it is located outside the microhomology (Figure 2B, C). Intriguingly, POLQ (QM1) more efficiently bypassed an AP site on the TMEJ substrates compared to primer-template fully annealed substrates (Figure 2B, C). Interestingly, an AP site located within the microhomology region inhibited POLQ (QM1) activity. POLQ (QM1) was not able to extend DNA at all from an AP site located at the 3’ end of the primer but was able to extend the undamaged opposite strand (Figure 2D), indicating that POLQ (QM1) promoted annealing the substrate (P_AP1/T). An AP site located within the microhomology strongly inhibited POLQ (QM1) DNA extension on both strands, indicating POLQ (QM1) hardly processed these substrates (P_AP3/T and P_AP5/T) (Figure 2E, F).

POLQ has been shown to efficiently incorporate A opposite an AP site on a primer-template fully annealed substrates, but the efficiency of T incorporation was about 100-fold lower (26,52). We showed that POLQ (QM1) promoted end-joining even when only T was provided for AP site bypass on the A-tailed TMEJ substrates (Supplementary Figure S6). As we expected, the AP site bypass efficiencies with T incorporation (Supplementary Figure S6B, C) were lower than the bypass efficiencies with A incorporation (Figure 2B,C). Similar to the T-tailed TMEJ substrates, an AP site located within the microhomology on the A-tailed TMEJ substrates strongly inhibited POLQ (QM1) activity (Supplementary Figure S6D–F); POLQ (QM1) more efficiently bypassed an AP site on the A-tailed TMEJ substrates compared to primer-template fully annealed substrates (Supplementary Figure S6B, C). For the A-tailed TMEJ substrates, DNA synthesis from the template strand (TA) was not analyzed because it was self-annealed and extended by POLQ (QM1) (Supplementary Figure S8, lane 8)

**Figure 3.**
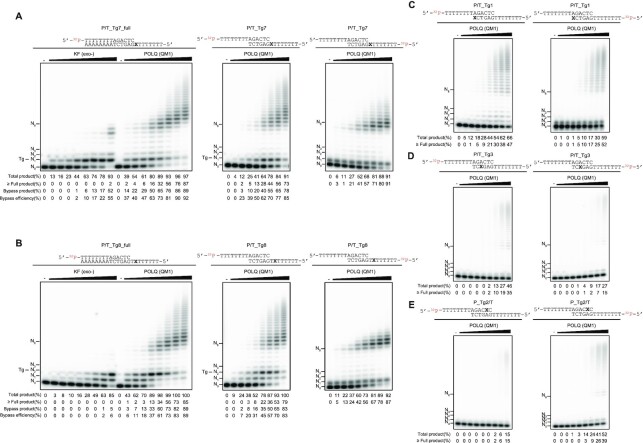
POLQ (QM1) efficiently bypasses a Tg on TMEJ substrates. Increasing concentrations of POLQ (QM1) (0, 0.3125, 0.625, 1.25, 2.5, 5, 10, 20, 40 nM) or KF (exo-) (0, 3.9, 7.8, 15.6, 31.2, 62.5, 125, 250, 500 fM) were incubated with primer-template fully annealed substrates or T-tailed TMEJ substrates carrying a Tg at different positions denoted as X (**A–E**). Expected substrates after synapsis formation and the strands labeled with ^32^P are shown above each gel image (A–E). All reaction mixtures had 100 nM substrate and 5 μM dATP, and were incubated at 37°C for 20 min. Locations of unreacted end-labeled primer (N_0_), template base position (N_1_, N_2_, N_3_), full-length product (N_8_) are labeled on each gel. The percentage (%) of total product was calculated as [≥ N_1_]/[≥ N_0_]. The percentage of fully and further extended product (≥ Full) was calculated as [≥N_8_]/[≥N_0_]. The percentage of bypass product for DNA damage at position N was defined as [≥N + 1]/[≥N_0_]. The bypass efficiency was calculated as [the percentage of bypass product]/[the percentage of total product]. These percentages are shown below each lane. Original gels are presented in Supplementary Figure S10.

Tg is one of the major DNA lesions formed by ionizing radiation and is a strong block to DNA polymerases (53). POLQ is known to bypass a Tg on fully annealed substrates (26,27) but whether it can bypass a Tg on TMEJ substrates is currently not known. Consistent with previous reports, POLQ (QM1) efficiently bypassed a Tg on fully annealed primer-template substrates, while KF (exo-) was not able to bypass Tg (Figure 3A, B). Similar to TMEJ substrates carrying an AP site, POLQ (QM1) bypassed a Tg efficiently when it was located outside the microhomology (Figure 3A, B). Although POLQ (QM1) was not able to extend DNA from an AP site at the end of primer (Figure 2D), it was able to extend DNA from a 3’ terminus with a Tg residue (Figure 3C). A Tg located within the microhomology inhibited POLQ (QM1) DNA extension on both strands (P/T_Tg3 and P_Tg2/T) (Figure 3D, E). Products larger than the fully extended products (>N_8_) were detected in Figures 2 and 3. However, these were not the products of terminal deoxynucleotidyl transferase (TdT) activity. Fully extended products were detached from the template, self-annealed between consecutive T and A sequences, and further extended by POLQ (QM1) (Supplementary Figure S8, lanes 9-12).

**Figure 4.**
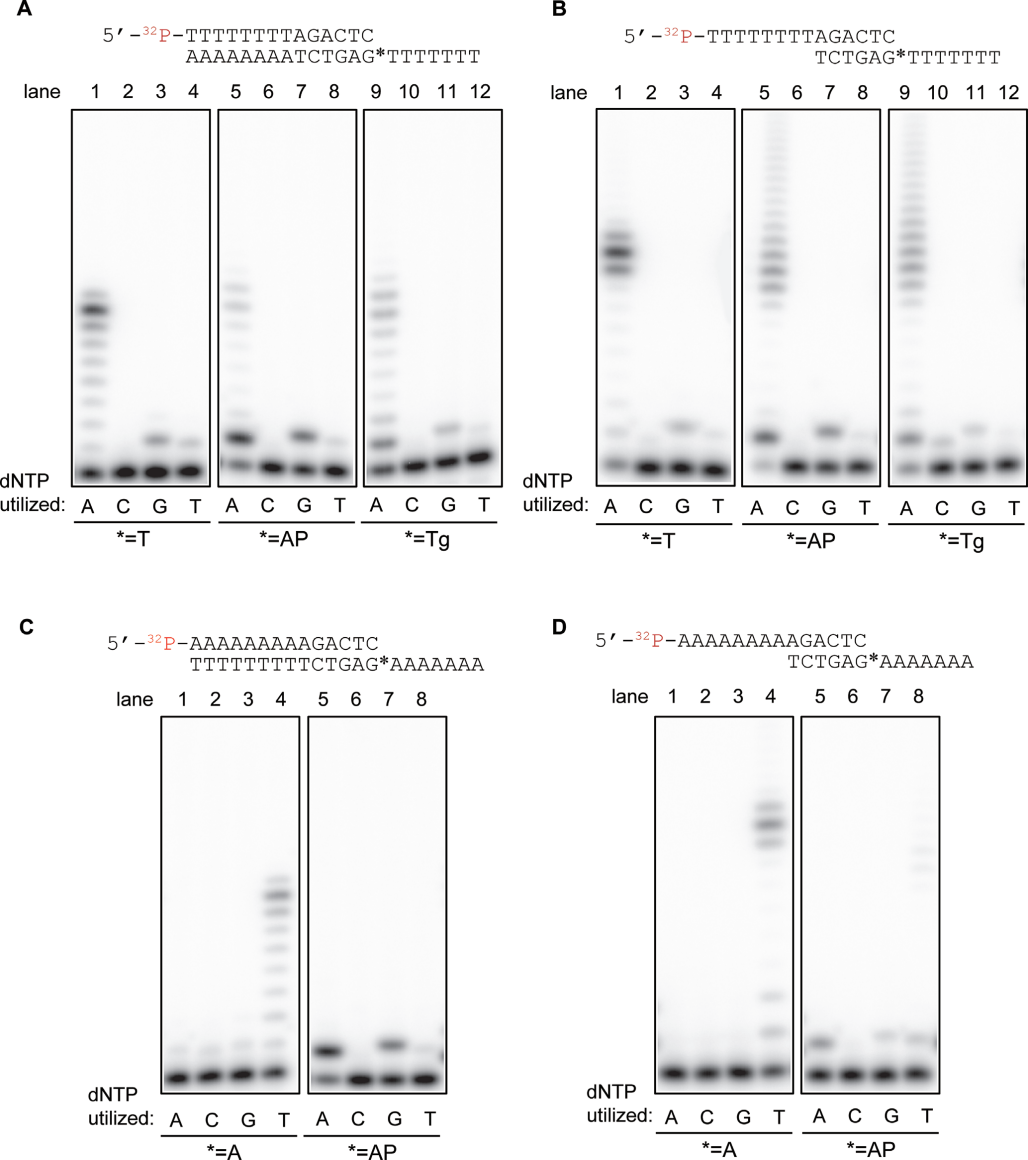
Nucleotide selectivity of POLQ. 1.25 nM (**A**, **C**) or 10 nM (**B, D**) POLQ (QM1) was incubated with 100 nM of a 5′-^32^P-labeled 14-mer primer annealed to a 22-mer (A, C) or a 14-mer (B, D) oligo in the presence of one of the indicated dNTPs (5 μM) for 20 min at 37°C. The first template base denoted by * was T, A, AP site (THF), or Tg. Original gels are presented in Supplementary Figure S10.

We then analyzed the nucleotide preference for the bypass of an AP site or Tg on TMEJ substrates (Figure 4). On fully annealed substrates, POLQ (QM1) efficiently incorporated A opposite an AP site, G and T were also inserted to a lesser degree, and C incorporation was too rare to be detected (Figure 4A, C, lanes 5–8). The results were consistent with previously published results (26,52). The tendency of nucleotide incorporation against an AP site on TMEJ substrates was similar, with A being most efficiently incorporated opposite an AP site (Figure 4B, D, lanes 5–8). POLQ (QM1) also efficiently incorporated A opposite a Tg lesion on both fully annealed (Figure 4A, lanes 9–12) and TMEJ substrates (Figure 4B, lanes 9–12).

### An AP site or Tg located within the microhomology inhibits the synapsis formation activity of POLQ

**Figure 5.**
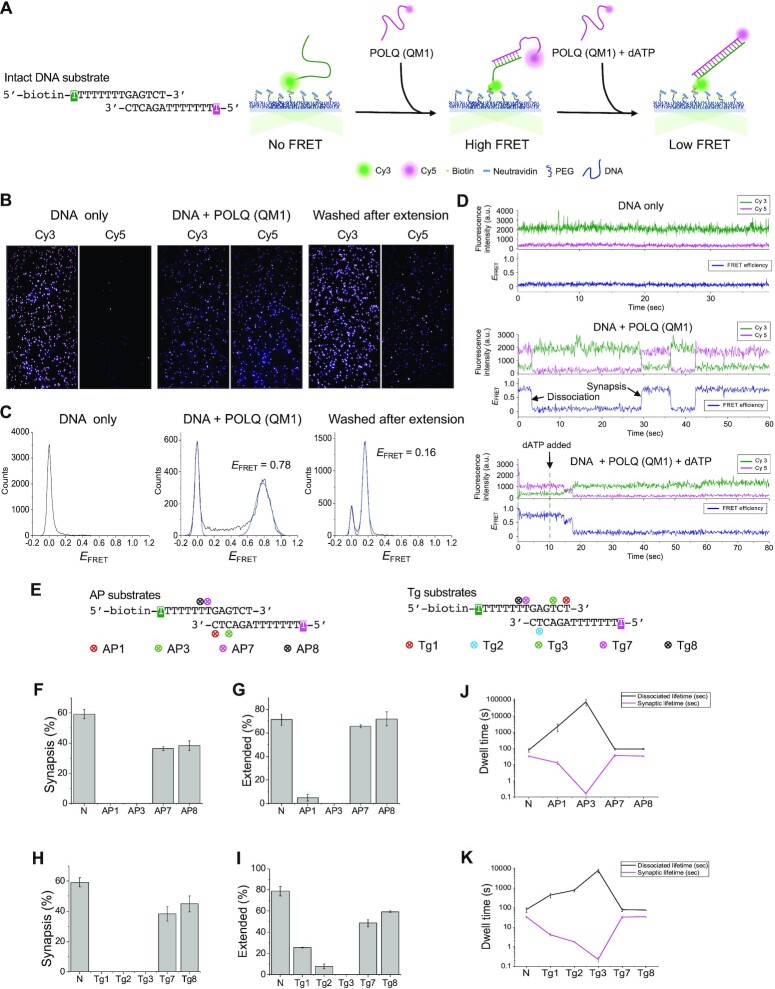
Single-molecule measurements of DNA synapsis formation and extension. (**A**) The experimental scheme of smFRET measurement shows synapse formation between a surface-immobilized Cy3-labeled strand and a Cy5-labeled strand by POLQ (QM1), and subsequent DNA extension of it upon the addition of dATP. Sizes of the glowing dyes reflect their expected fluorescence signals along the process. (**B**) Representative fluorescence images of the donor and acceptor channels at the following steps: surface-bound Cy3-labeled strand with Cy5-labeled strand in solution (left), POLQ (QM1) added (middle), and POLQ (QM1) washed away after extension (right). (**C**) FRET histograms measured at the steps in (B). *E*_FRET_ values of the non-zero FRET peaks are noted. (**D**) Representative single-molecule traces of donor fluorescence (green), acceptor fluorescence (magenta), and *E*_FRET_ (navy) at the steps in (B). For the extension step, the traces were shown from the moment of flowing in dATP (arrow). (**E**) Design of DNA substrates with an AP site or Tg lesion modification. The locations of the lesion are cross-marked and the labeling sites for fluorescent dyes are highlighted in green (Cy3) and magenta (Cy5). (**F**–**I**) Relative population of synapsis or extended DNA molecules for DNA substrates with a lesion compared to that of the intact DNA substrate (N). Error bars represent the standard error of the mean (SEM) from triplicate measurements. (**J**, **K**) Synaptic (high FRET) and dissociated (zero FRET) lifetimes for DNA substrates with an AP site (J) or Tg (K) compared to those of the intact DNA substrate, shown in log scale. Mean and SEM were obtained from three concatenated traces by the sHaRPer method.

To investigate the mechanism of how an AP site or a Tg lesion located within or near the microhomology region inhibits POLQ (QM1) activity (Figures 2 and 3), we developed a single molecule Förster resonance energy transfer (smFRET) assay. One oligonucleotide strand was biotinylated and labeled with the donor dye, Cy3, and attached to the PEG-coated surface via neutravidin (Figure 5A). The other strand was labeled with the acceptor dye, Cy5, and added to the solution to observe the annealing to the Cy3-labeled strand by POLQ (QM1). An AP site or a Tg lesion was introduced at varying positions of either strand (Figure 5B). When the Cy5-labeled strand was added to the immobilized Cy3-labeled strand at 40 nM, two undamaged strands did not undergo synapsis formation spontaneously, as indicated by the absence of fluorescent spots in the Cy5 channel and negligible high FRET population (Figure 5B,C). Upon addition of POLQ (QM1), the two strands formed a bound pair, as indicated by emerging Cy5 spots and a corresponding population at FRET efficiency, *E*_FRET_ = 0.78 (Figure 5B, C). The synapse formation was reversible as the FRET traces showed frequent transitions between the synaptic and dissociated states (Figure 5D). After 10 min incubation of POLQ (QM1) with 5 μM dATP, the FRET population at 0.78 disappeared and a new population appeared at *E*_FRET_ = 0.16, which we interpreted as representing fully extended DNA pairs as the rigidity of the duplex DNA leads to an increased distance between the Cy3 and Cy5 dyes (Figure 5B, C). During the extension, DNA pairs exhibited a stepwise decrease in FRET efficiency and eventually transitioned to a stable low FRET state (Figure 5D). In contrast, the high and low FRET states were not detected when the TMEJ substrate was incubated with KF (exo-) (Supplementary Figure S7A), indicating that KF (exo-) was not able to induce synapsis formation and extend the TMEJ substrate. On the other hand, when the fully annealed standard substrate was incubated with POLQ (QM1) or KF (exo-) in the presence of dATP, stable low FRET states were detected in both cases (Supplementary Figure S7B), indicating that both POLQ (QM1) and KF (exo-) were able to extend the fully annealed standard substrate in this assay system.

The effect of a lesion on the synapsis formation and extension activities was quantified by comparing the relative FRET populations at both stages for oligonucleotide pairs with the lesions at varying positions (Figure 5F–I and Supplementary Figure S9). Consistent with the sequencing gel analysis, the effect of a lesion strongly depended on its position; a lesion closer to the center of the microhomology region inhibited synapsis formation and polymerization to a greater extent for both AP sites and Tg lesions. A lesion located outside the microhomology region (AP7, AP8, Tg7 and Tg8) had only a minor effect on synapsis formation and extension (Figure 5F–I). After extension, the FRET efficiency of the damaged DNA pairs was higher than that of the undamaged DNA pair, ranging from 0.15 to 0.38, likely due to the less rigid helical structure of DNA with a lesion. (Supplementary Figure S9B, D). Even though the synapsis products with a lesion in the microhomology region were virtually not observed, we were able to measure the lifetime of the synaptic and dissociated states from the rare synapsis events. As the annealing event was extremely rare for the critical lesions, we applied the sHaRPer method to create ∼4 × 10^6^ s long concatenated traces (54). Dwell times obtained from the concatenated traces revealed dramatic dependence on the lesion position, with the longest dissociated lifetimes being 7.3 × 10^4^ ± 3 × 10^4^ s and 7.7 × 10^3^ ± 1.6 × 10^3^ s for AP3 and Tg3, respectively, while the dissociated lifetime for the non-damaged DNA was 77 ± 20 s (Figure 5J, K). At the same time, the lifetimes of the synaptic state were much shorter for DNA with a lesion in the microhomology region, showing a symmetrical trend to that of the dissociated lifetime, which indicates the thermal instability of the synapsis products with a lesion in the microhomology region. We found that overall, an AP site is more inhibitory than a Tg lesion as the synaptic and dissociated lifetimes showed a larger spread for the substrates with an AP site (Figure 5J, K).

One possible limitation of our study is that we used a truncated version of POLQ (QM1) that is missing the N-terminus HLD domain. It has been shown that the HLD is able to stably bridge noncomplementary single DNA strands (42), and it is therefore possible that full-length POLQ may anneal microhomologies carrying an AP site or Tg more efficiently than POLQ (QM1).

## DISCUSSION

SOBP carbon ions have been used for heavy ion radiation therapy to efficiently kill tumor cells while minimizing biological effects on surrounding normal tissues by utilizing the Bragg peak (55). Heavy ion radiation is known to induce complex DSBs, which contain additional DNA damage such as AP sites, oxidative base damage, or SSBs, near DSBs (12). In this study, we provide the evidence that TMEJ has an important role in processing hiLET-DSBs. *POLQ*^−/−^ clones showed a lower level of survival and higher frequency of micronuclei and chromatid breaks with carbon ions than with X-rays, indicating that POLQ is important to process hiLET-DSBs (Figure 1). We note that we were able to conduct the chromosome assay only once due to the limited access to the synchrotron. Two independent *POLQ*^−/−^ clones served as biological replicates and showed similar characteristics in all the experiments we performed including the chromosome assay.

In a previous study in CHO cells, the RBE values of high LET to low LET were >1.00 in HR-deficient cells (*XRCC2*^−/−^ or *XRCC3*^−/−^) but not in NHEJ-deficient cells (*XRCC4*^−/−^) (48). Furthermore, it has been shown that complex DSBs are hardly processed by NHEJ (9–11). In our study, the RBE values were greater than 1.00 in *POLQ*^−/−^ cells. Resection-mediated DSB repair, including TMEJ and HR is therefore thought to be important to process hiLET-DSBs.

Our biochemical assays revealed that POLQ can perform TLS across an AP site and a Tg during end-joining when these lesions were located outside the microhomology (Figures 2 and 3). Interestingly, we observed higher bypass efficiency of AP sites on TMEJ substrates compared to the corresponding fully annealed substrates (Figure 2 and Supplementary Figure S6). The degree of DNA distortion caused by AP sites may be different in TMEJ and fully annealed substrates and this may influence the bypassing activity of POLQ. We found that an AP site or a Tg located within the microhomology strongly inhibits POLQ’s synapsis formation activity and the DNA in these substrates was therefore hardly extended. When an AP site or Tg was located at the end of the primer, the annealing step was moderately inhibited while DNA extension was completely inhibited for an AP site but not for Tg. On the other hand, when these lesions were located outside the microhomology, POLQ efficiently annealed these substrates and extended DNA (Figures 2-5).

**Figure 6.**
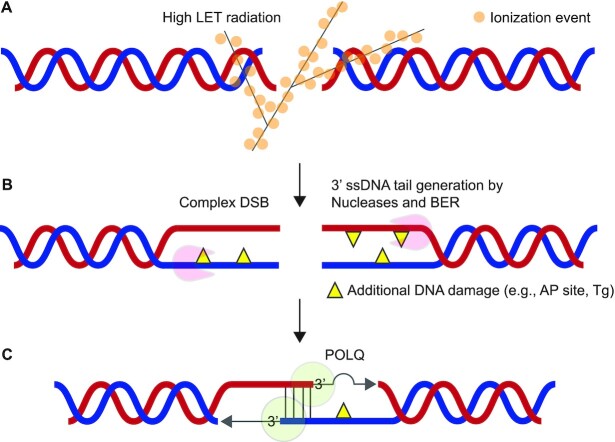
A model of TMEJ-mediated repair following high LET radiation. (**A**) High LET radiation causes complex DSBs, which carry additional DNA damage in close proximity to the DSB sites. Since base damage near DSB inhibits NHEJ, the majority of complex DSBs are repaired after DNA end resection (12). (**B**) SSBs generated close to DSBs or nicks originating from oxidative base damage by BER may alleviate the need for the step of nick introduction by the MRE11/CtIP endonuclease and directly trigger resection by the exonuclease activity of EXO1 or MRE11/CtIP (12). The endonuclease activity of ARTEMIS, which is dependent on DNA-PKcs and/or 5’-3’ exonuclease activity of ARTEMIS, which is not dependent on DNA-PKcs (59), may also resect complex DSB ends (21). (**C**) POLQ promotes synapsis formation of the two resected 3’-ssDNA tails and efficiently bypasses oxidative DNA damage located on the tails. An AP site or Tg located within the microhomology inhibits the synapsis formation activity of POLQ. These lesions may be removed by AP endonuclease, NEIL3, or NEIL1, which are active on ssDNA tails (60–62).

The majority of complex DSBs are repaired by resection-mediated pathways (18–21). Following DNA end resection, POLQ can promote end-joining when DNA lesions are located outside the microhomology. Complex DSBs that POLQ cannot process may be repaired by HR or TMEJ again after DNA damage near break sites is removed by nucleases. A model for POLQ-mediated repair of complex DSBs is shown in Figure 6.

It has been proposed that after high LET radiation, some relatively ‘clean’ DSBs are joined by NHEJ but the other DSBs are processed by resection-mediated pathways even in G_1_ phase cells (18–20). Resected DSBs in G_1_ cells cannot be processed by either HR or NHEJ because of the absence of homologous sister chromatid as a repair template for HR and resected DSBs block KU loading (56). POLQ functions in M phase cells (57), but it may also function in the G_1_ phase cells to process resected complex DSBs. It has been reported that POLQ protein loaded on chromatin is enriched in the G_1_ phase (58). This assumption will be important to address in future studies.

## DATA AVAILABILITY

The authors confirm that all relevant data are present in the manuscript and its supplementary data. Materials in this study are available from the corresponding author upon reasonable request.

## Supplementary Material

gkad076_Supplemental_File
