# Supplementary material for: DNA polymerase θ-mediated repair of high LET radiation-induced complex DNA double-strand breaks
Source: Nucleic Acids Res. 2023 Feb 20;51(5):2257–69. doi: 10.1093/nar/gkad076 (PMC10018357; doi:10.1093/nar/gkad076)
Supplement: gkad076_Supplemental_File [file gkad076_supplemental_file.pdf]

# DNA polymerase θ-mediated repair of high LET radiation-induced complex DNA double-strand breaks

Geunil Yi, Yubin Sung, Chanwoo Kim, Jae Sun Ra, Hirokazu Hirakawa,
Takamitsu A Kato, Akira Fujimori, Hajin Kim, and Kei-ichi Takata

## Supplementary Data

Correspondence: Kei-ichi Takata
Center for Genomic Integrity, Institute for Basic Science (IBS)
Ulsan 44919, Republic of Korea
Phone: +82-52-217-5536
E-mail: ktakata@ibs.re.kr

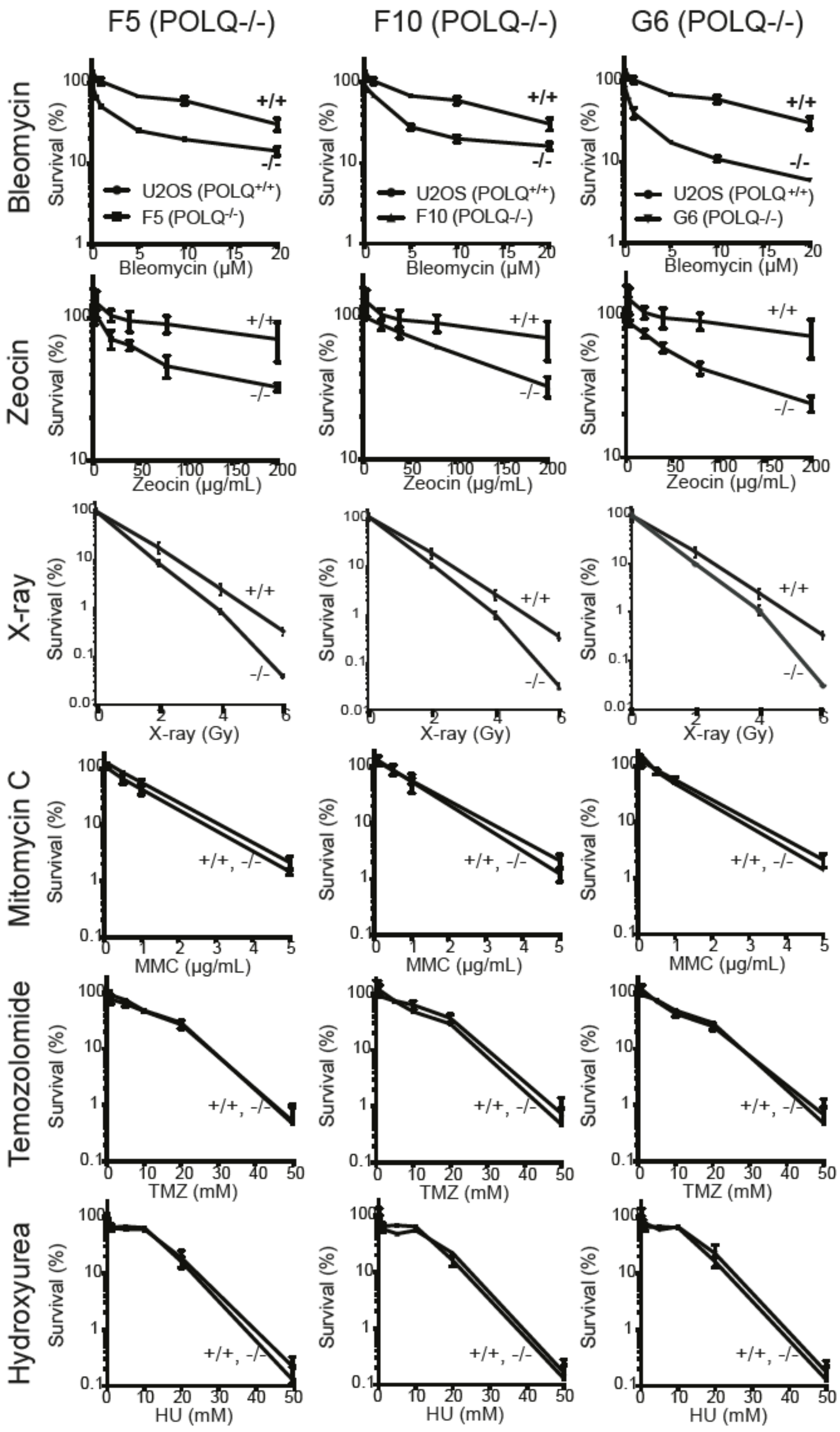


**Supplementary Figure S1**

**Supplementary Figure S1.** *POLQ*-knockout human cell lines are sensitive to DNA double-strand break-inducing agents. Three *POLQ*$^{-/-}$ U2OS cell lines, F5, F10, and G6, and their parental U2OS cell line were exposed to the indicated doses of bleomycin for 144 h, Zeocin for 144 h, mitomycin C for 48 h, temozolomide for 48 h, hydroxyurea for 48 h, and x-rays. Viability was determined by measuring ATP content except for x-rays. Viability for x-rays was determined by clonogenic assay. The mean of three independent experiments is shown, with S.D. indicated by error bars.

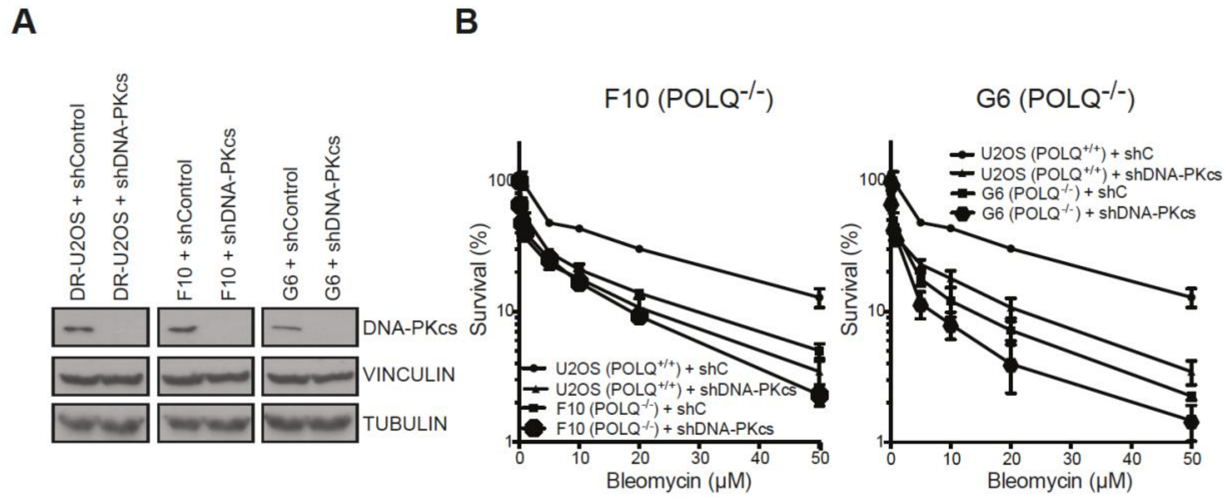


**Supplementary Figure S2.** DNA-PKcs inhibition in *POLQ*-knockout cells leads to increased DSB sensitivity. (**A**) Immunoblot showing efficacy of shRNA-mediated knockdown of DNA-PKcs in *POLQ*$^{+/+}$ and two *POLQ*$^{-/-}$ (F10 and G6) cells. shControl (shC) served as a control, and α-Tubulin and Vinculin served as the loading control. (**B**) The cells were exposed to the indicated doses of bleomycin for 144 h. Viability was determined by measuring ATP content. The mean of three independent experiments is shown, with S.D. indicated by error bars.

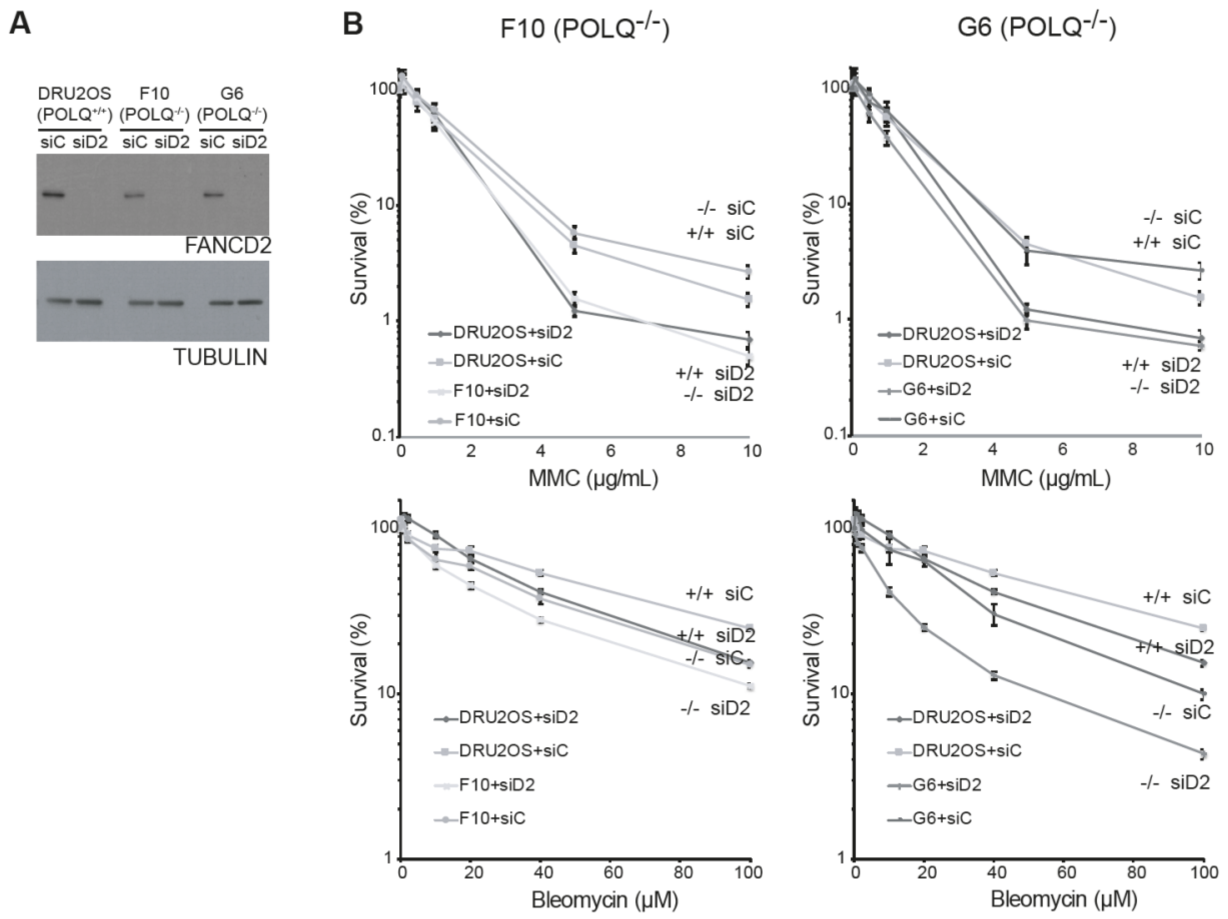


**Supplementary Figure S3.** POLQ does not influence ICL sensitivity. (**A**) Immunoblot showing the efficacy of siRNA-mediated knockdown of FANCD2 (siD2) in *POLQ*$^{+/+}$ and two *POLQ*$^{-/-}$ (F10 and G6) cell lines. siC served as a negative control and α-Tubulin as a loading control. (**B**) The cells were exposed to indicated doses of bleomycin for 48 h or mitomycin C for 48 h. Viability was determined by measuring ATP content. FANCD2-depleted *POLQ*$^{-/-}$ cells were the most sensitive to bleomycin and were more sensitive than mock-depleted *POLQ*$^{-/-}$ or FANCD2-depleted *POLQ*$^{+/+}$ cells. The mean of three independent experiments is shown, with S.D. indicated by error bars.

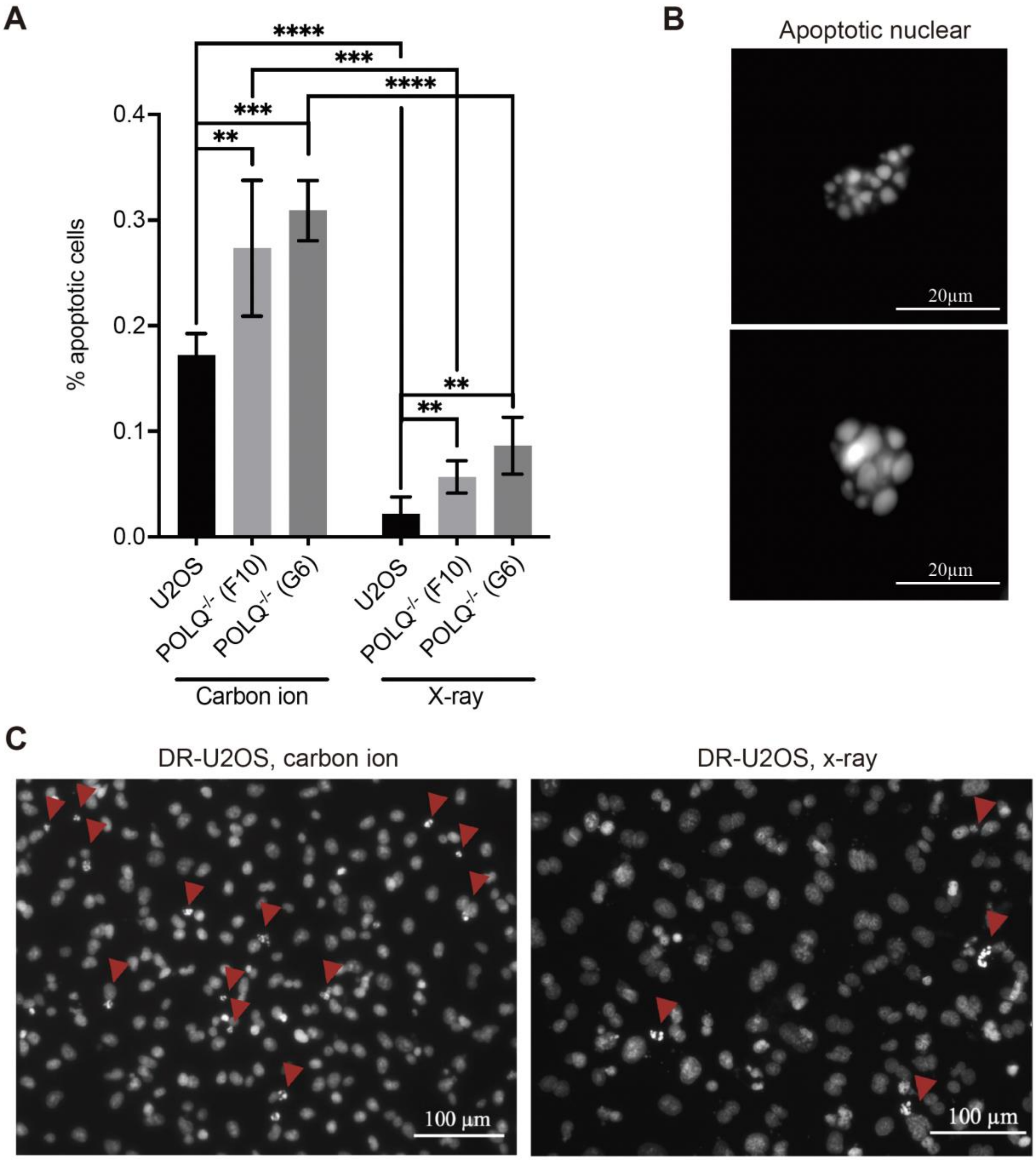


**Supplementary Figure S4**

**Supplementary Figure S4.** Apoptosis is induced more by carbon ions than x-rays. **(A**) DAPI-stained images and quantification results of U2OS cells after irradiation. Percentage of apoptotic cells among binucleated *POLQ*-proficient and deficient cells irradiated with 2 Gy of either carbon ions or x-rays. (**B**) Representative magnified cell images undergoing apoptosis. (**C**) Representative images of cells irradiated with carbon ions or x-rays. Arrowheads show cells undergoing apoptosis.

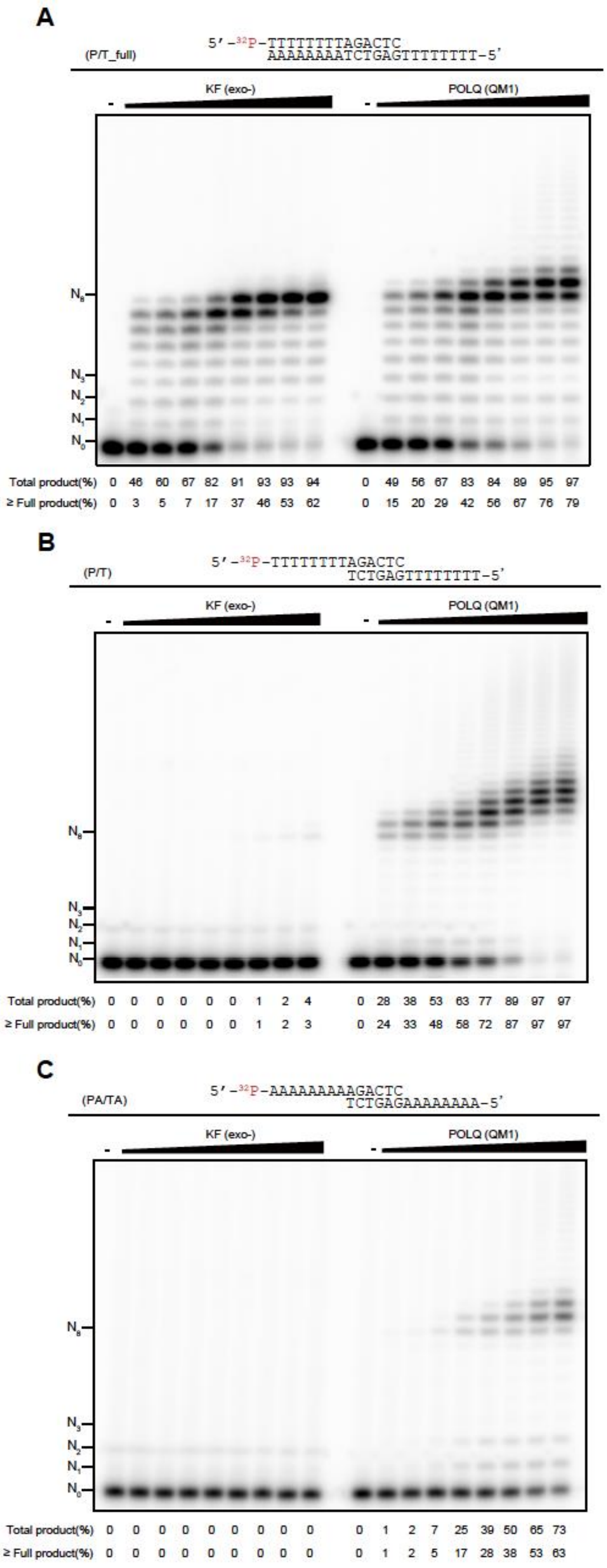


**Supplementary Figure S5**

**Supplementary Figure S5.** The unique microhomology-mediated end-joining activity of POLQ. Increasing concentrations of POLQ (QM1) (0, 0.3125, 0.625, 1.25, 2.5, 5, 10, 20, 40 nM) or KF (exo-) (0, 3.9, 7.8, 15.6, 31.2, 62.5, 125, 250, 500 fM) were incubated with primer-template fully annealed substrates (**A**), T-tailed TMEJ substrates (**B**), or A-tailed TMEJ substrates (**C**). Expected substrates after annealing and the strands labeled with $^{32}P$ are shown above each gel image. All reaction mixtures had 100 nM substrate and 5 µM dTTP and were incubated at 37 °C for 20 min. Locations of unreacted end-labeled primer ($N_0$), template base position ($N_1$, $N_2$, $N_3$), and full-length product ($N_8$) are shown. The percentage (%) of total product was calculated as [≥ $N_1$]/[≥ $N_0$]. The percentage of fully and further extended product (≥ Full) was calculated as [≥ $N_8$]/[≥ $N_0$]. These percentages are shown below each lane.

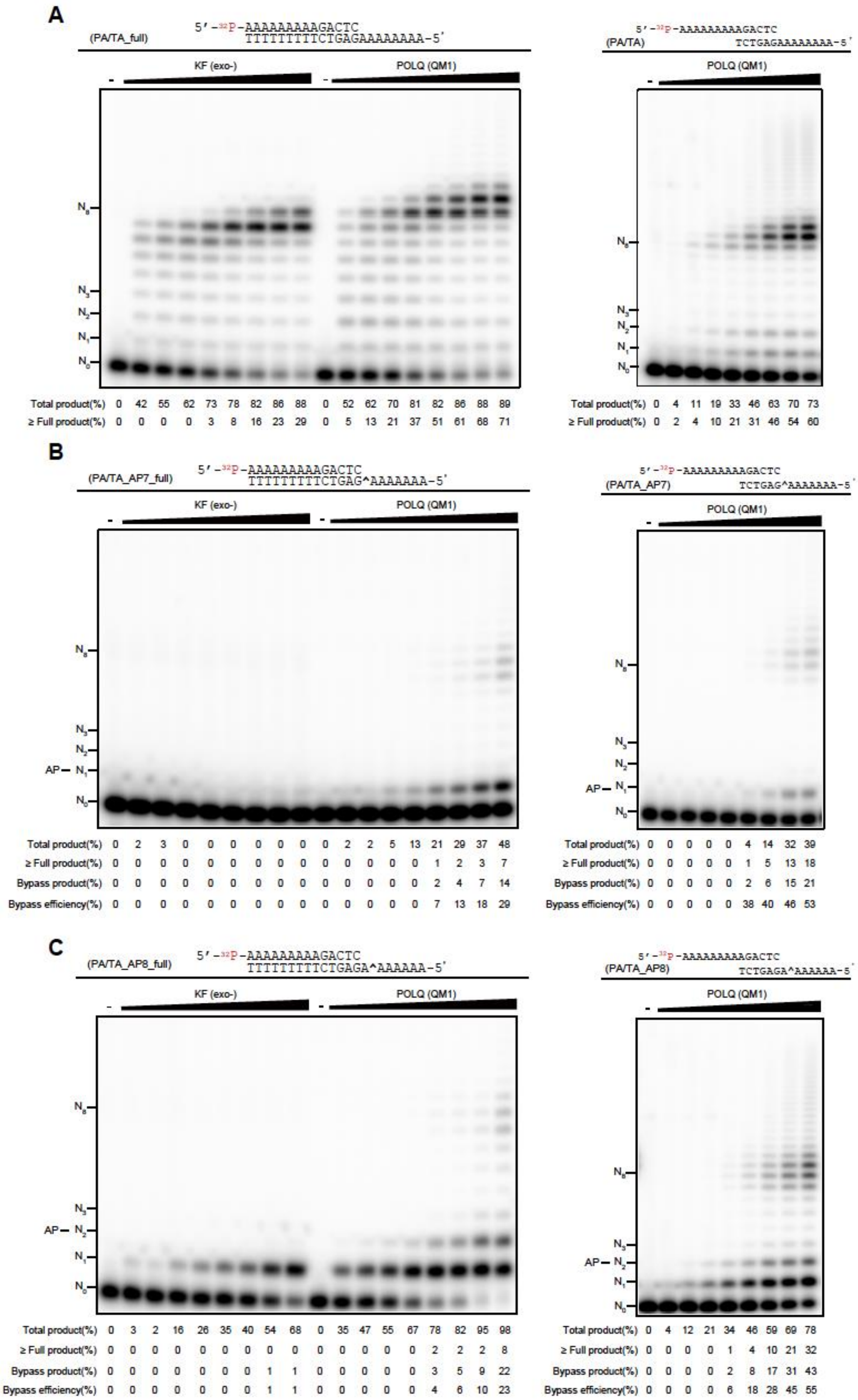
A
(PA/TA_full)
5′-32P-AAAAAAAAAGACTC
TTTTTTTTTCTGAGAAAAAAAAA-5′
KF (exo-)
POLQ (QM1)
N8
N3
N2
N1
N0
Total product(%) 0 42 55 62 73 78 82 86 88 0 52 62 70 81 82 86 88 89
≥ Full product(%) 0 0 0 0 3 8 16 23 29 0 5 13 21 37 51 61 68 71
(PA/TA)
5′-32P-AAAAAAAAAGACTC
TCTGAGAAAAAAAAA-5′
POLQ (QM1)
Total product(%) 0 4 11 19 33 46 63 70 73
≥ Full product(%) 0 2 4 10 21 31 46 54 60
B
(PA/TA_AP7_full)
5′-32P-AAAAAAAAAGACTC
TTTTTTTTTCTGAG^AAAAAAA-5′
KF (exo-)
POLQ (QM1)
AP
Total product(%) 0 2 3 0 0 0 0 0 0 0 2 2 5 13 21 29 37 48
≥ Full product(%) 0 0 0 0 0 0 0 0 0 0 0 0 0 0 1 2 3 7
Bypass product(%) 0 0 0 0 0 0 0 0 0 0 0 0 0 0 2 4 7 14
Bypass efficiency(%) 0 0 0 0 0 0 0 0 0 0 0 0 0 0 7 13 18 29
(PA/TA_AP7)
5′-32P-AAAAAAAAAGACTC
TCTGAG^AAAAAAA-5′
POLQ (QM1)
Total product(%) 0 0 0 0 0 4 14 32 39
≥ Full product(%) 0 0 0 0 0 1 5 13 18
Bypass product(%) 0 0 0 0 0 2 6 15 21
Bypass efficiency(%) 0 0 0 0 0 38 40 46 53
C
(PA/TA_AP8_full)
5′-32P-AAAAAAAAAGACTC
TTTTTTTTTCTGAGA^AAAAAA-5′
KF (exo-)
POLQ (QM1)
Total product(%) 0 3 2 16 26 35 40 54 68 0 35 47 55 67 78 82 95 98
≥ Full product(%) 0 0 0 0 0 0 0 0 0 0 0 0 0 0 2 2 2 8
Bypass product(%) 0 0 0 0 0 0 0 1 1 0 0 0 0 0 3 5 9 22
Bypass efficiency(%) 0 0 0 0 0 0 0 1 1 0 0 0 0 0 4 6 10 23
(PA/TA_AP8)
5′-32P-AAAAAAAAAGACTC
TCTGAGA^AAAAAA-5′
POLQ (QM1)
Total product(%) 0 4 12 21 34 46 59 69 78
≥ Full product(%) 0 0 0 0 1 4 10 21 32
Bypass product(%) 0 0 0 0 2 8 17 31 43
Bypass efficiency(%) 0 0 0 0 6 18 28 45 55

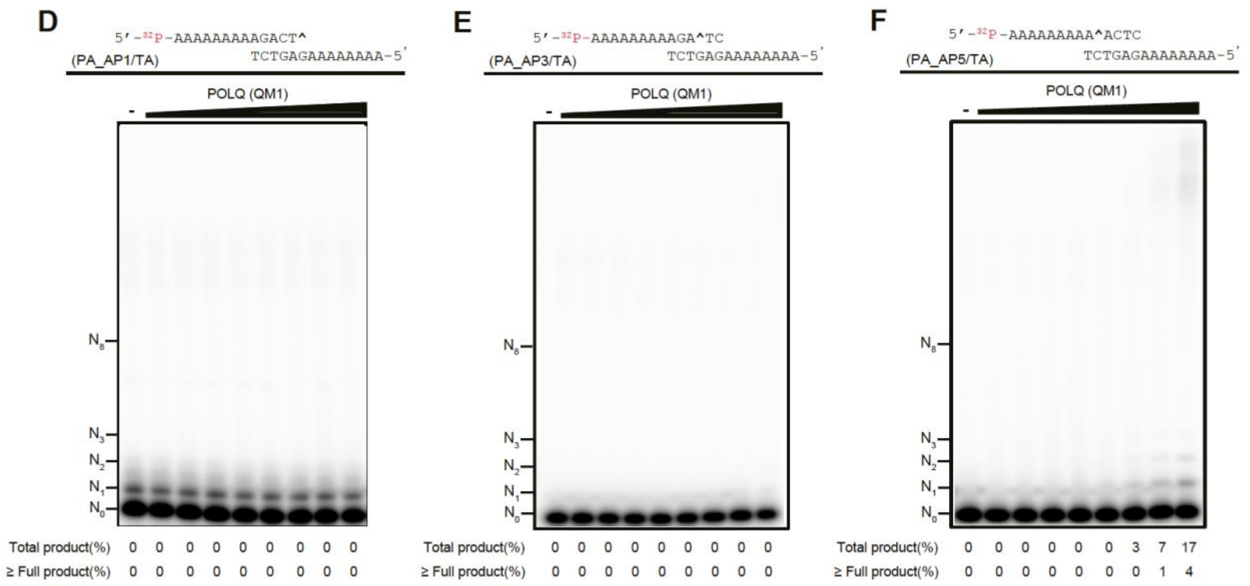


**Supplementary Figure S6.** POLQ (QM1) is able to insert T opposite an AP site and extend on TMEJ substrates. Increasing concentrations of POLQ (QM1) (0, 0.3125, 0.625, 1.25, 2.5, 5, 10, 20, 40 nM) or KF (exo-) (0, 3.9, 7.8, 15.6, 31.2, 62.5, 125, 250, 500 fM) were incubated with primer-template fully annealed substrates or A-tailed TMEJ substrates carrying no DNA damage (**A**) or an AP site at different positions denoted as ^ (**B-F**). Expected substrates after annealing and the strands labeled with $^{32}P$ are shown above each gel image. All reaction mixtures had 100 nM substrate and 5 µM dTTP and were incubated at 37 °C for 20 min. Locations of unreacted end-labeled primer ($N_0$), template base position ($N_1$, $N_2$, $N_3$), full-length product ($N_8$), and positions of THF on the template are shown as AP. The percentage (%) of total product was calculated as [≥ $N_1$]/[≥ $N_0$]. The percentage of fully and further extended product (≥ Full) was calculated as [≥ $N_8$]/[≥ $N_0$]. The percentage of bypass product for DNA damage at position N was defined as [≥ N + 1]/[≥ $N_0$]. The bypass probability was calculated as [the percentage of bypass product]/[the percentage of total product]. These percentages are shown below each lane.

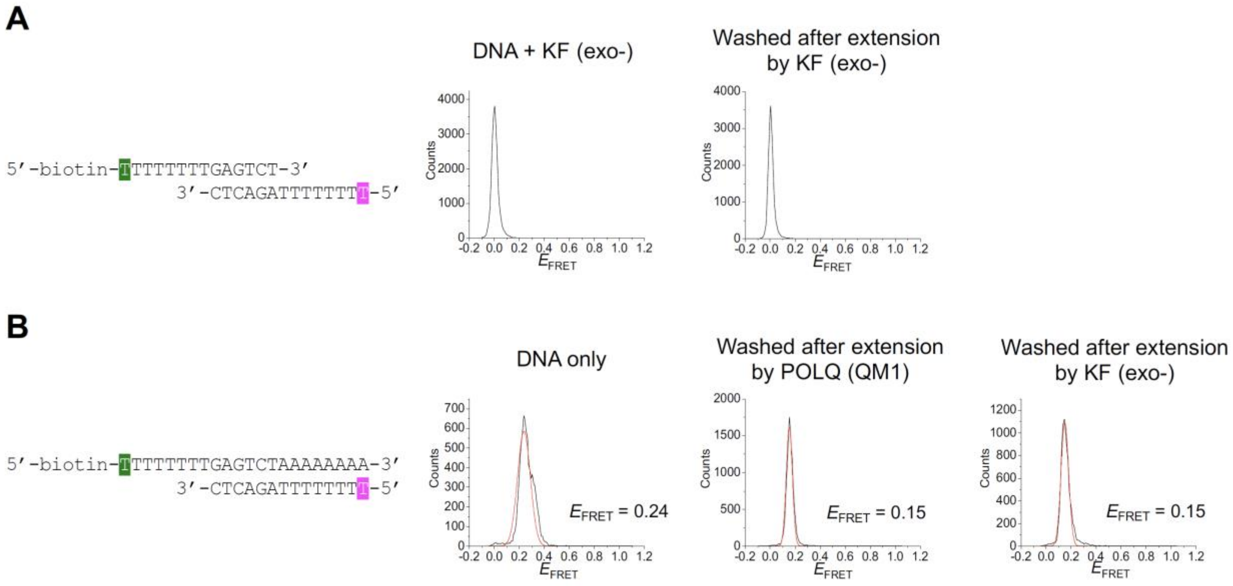


**Supplementary Figure S7.** Single-molecule measurements of DNA synapsis formation and extension. (**A**) FRET histograms of a TMEJ substrate synapsis formation by KF (exo-) (left) and after extending with dATP and washing away KF (exo-) (right). (**B**) FRET histograms of a fully annealed substrate before the reaction (left) and after extending with dATP and washing away POLQ (QM1) (middle) or KF (exo-) (right). EFRET values of the non-zero FRET peaks are noted.

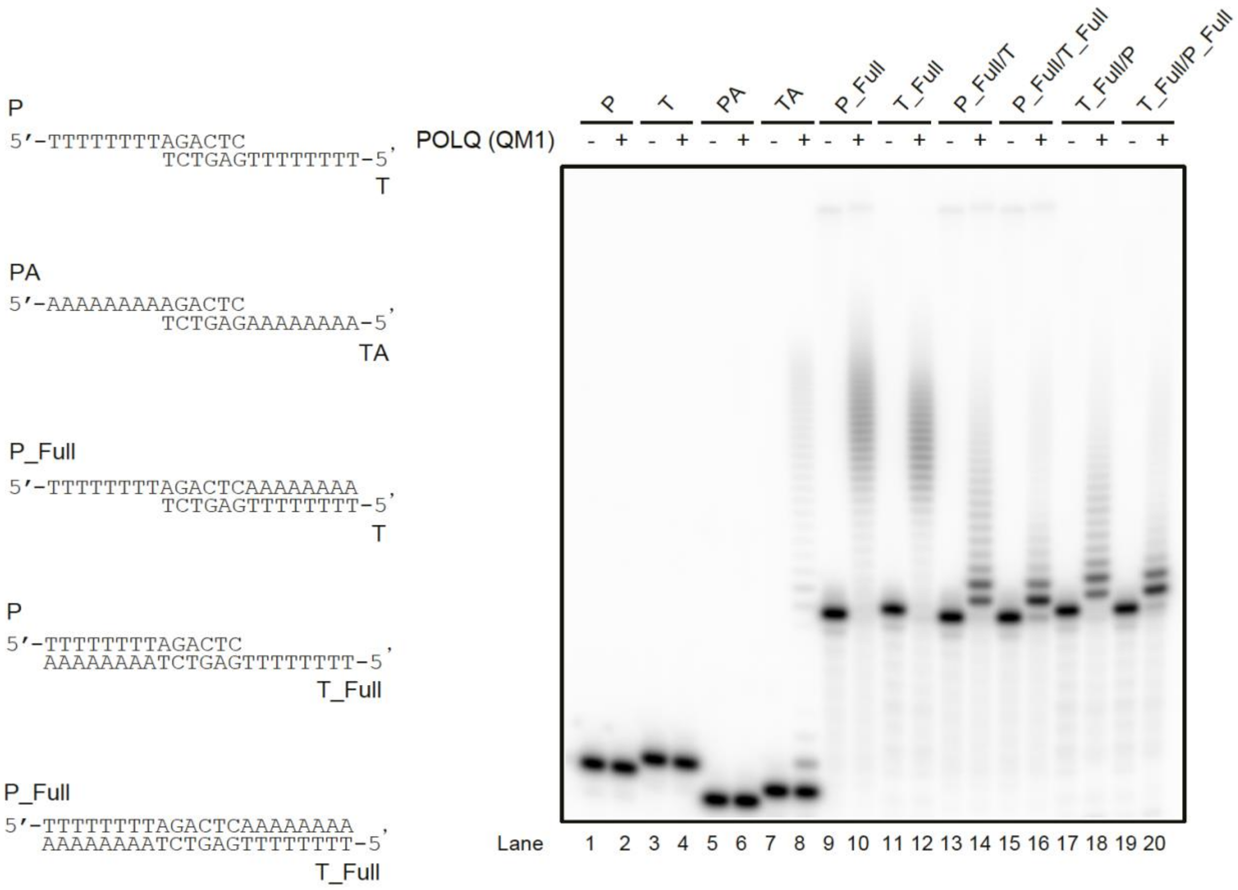


**Supplementary Figure S8.** Oligonucleotides used in this study were evaluated and further investigated. Substrates used in this study are shown on the left. Oligonucleotides shown above each lane were 5’-$^{32}$P labeled and tested whether they can be self-annealed and extended by POLQ (QM1) (lanes 1-20). Reaction mixtures included dATP for lanes 1-4 and 9-20 and dTTP for lanes 5-8 and were incubated with or without 40 nM POLQ (QM1) at 37 °C for 20 min.

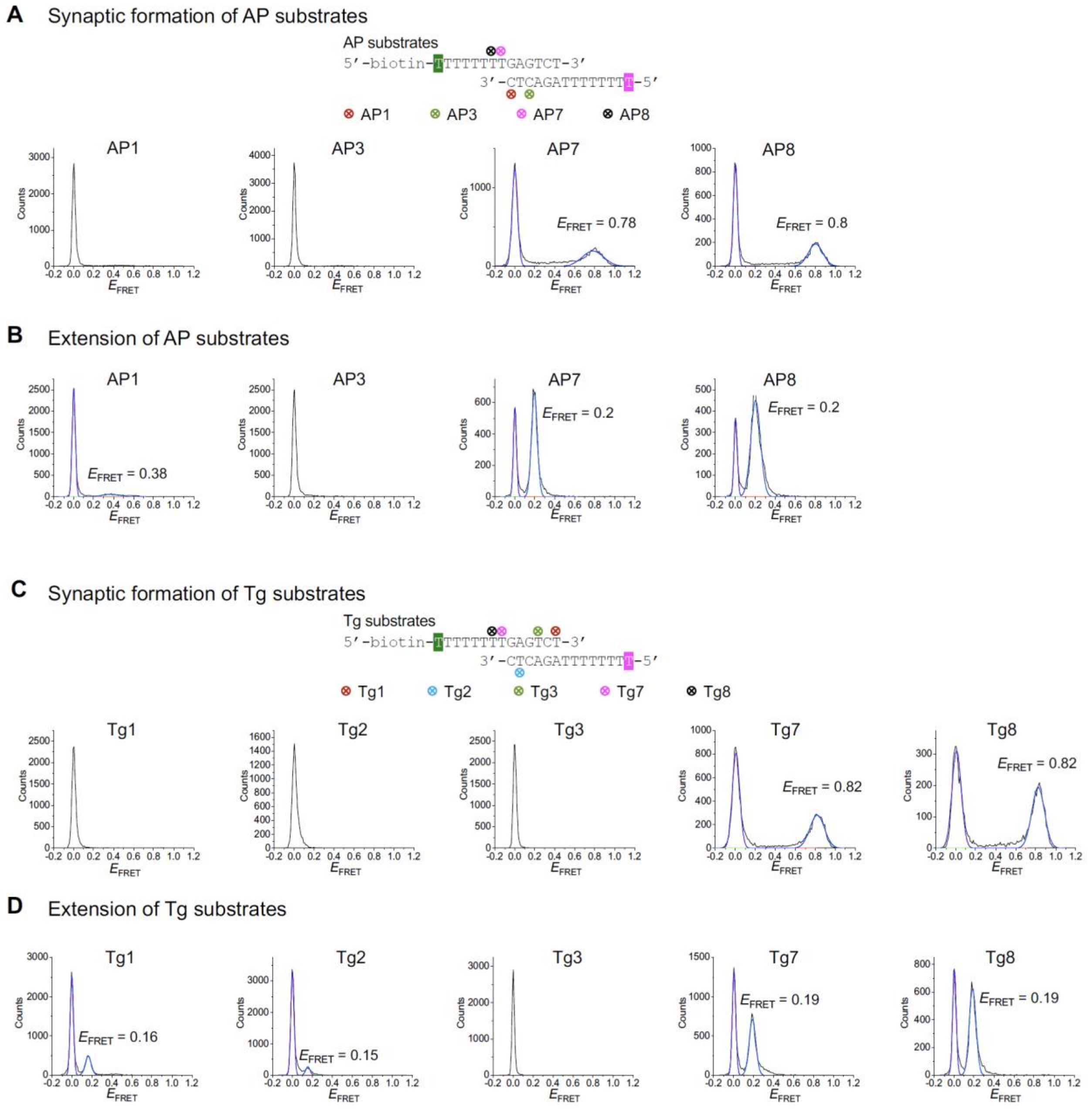


**Supplementary Figure S9.** Single-molecule measurements of translesion synthesis during TMEJ. FRET histograms of AP site and Tg substrates annealed by POLQ (QM1) (**A, C**) and after extending with dATP and washing away POLQ (QM1) (**B, D**). $E_{FRET}$ values of the non-zero FRET peaks are noted.

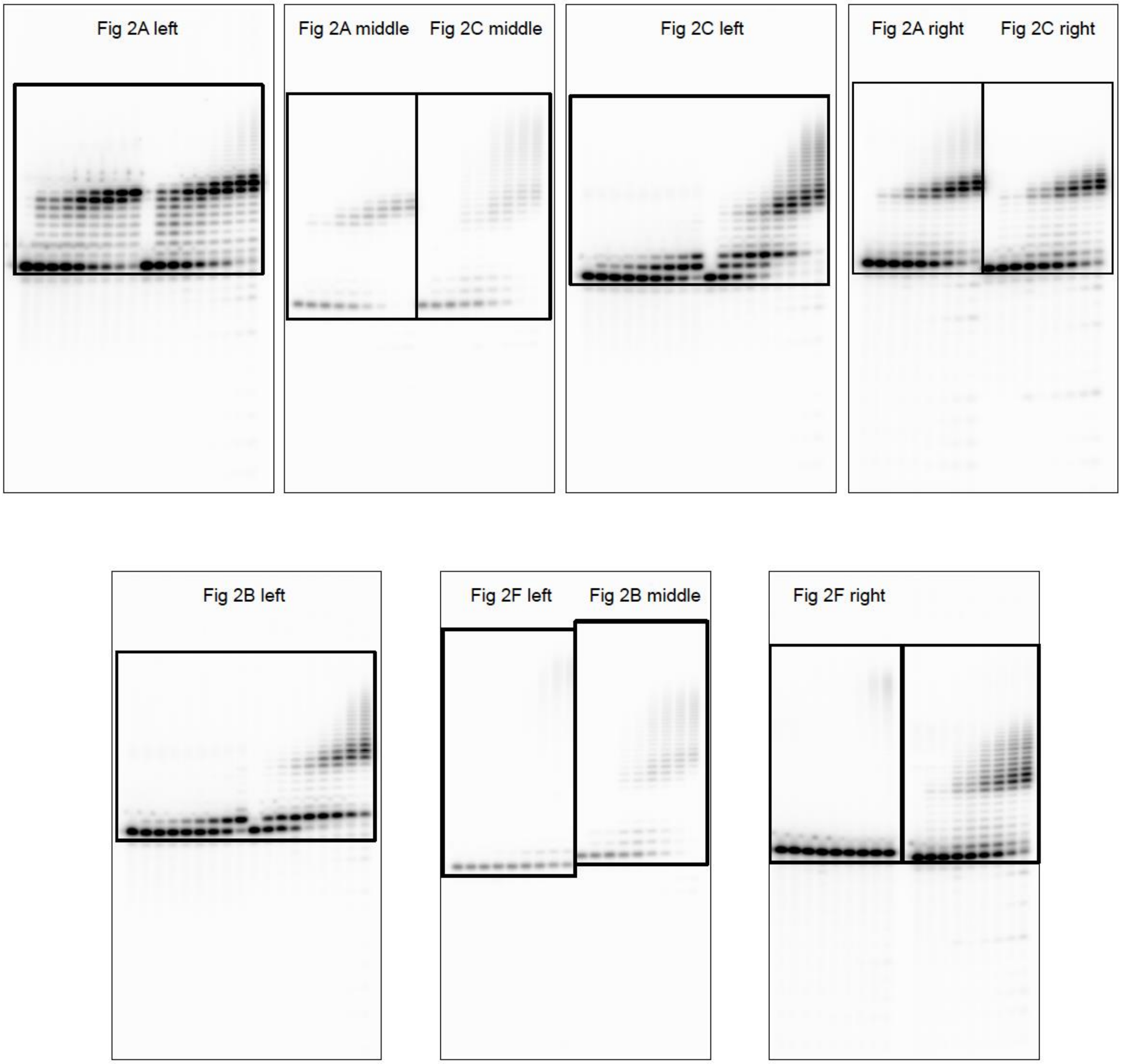
Fig 2A left
Fig 2A middle
Fig 2C middle
Fig 2C left
Fig 2A right
Fig 2C right
Fig 2B left
Fig 2F left
Fig 2B middle
Fig 2F right

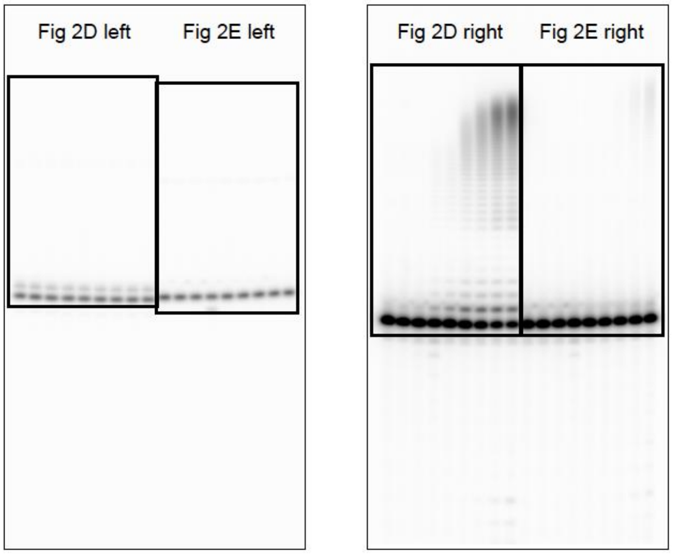
Fig 2D left
Fig 2E left
Fig 2D right
Fig 2E right

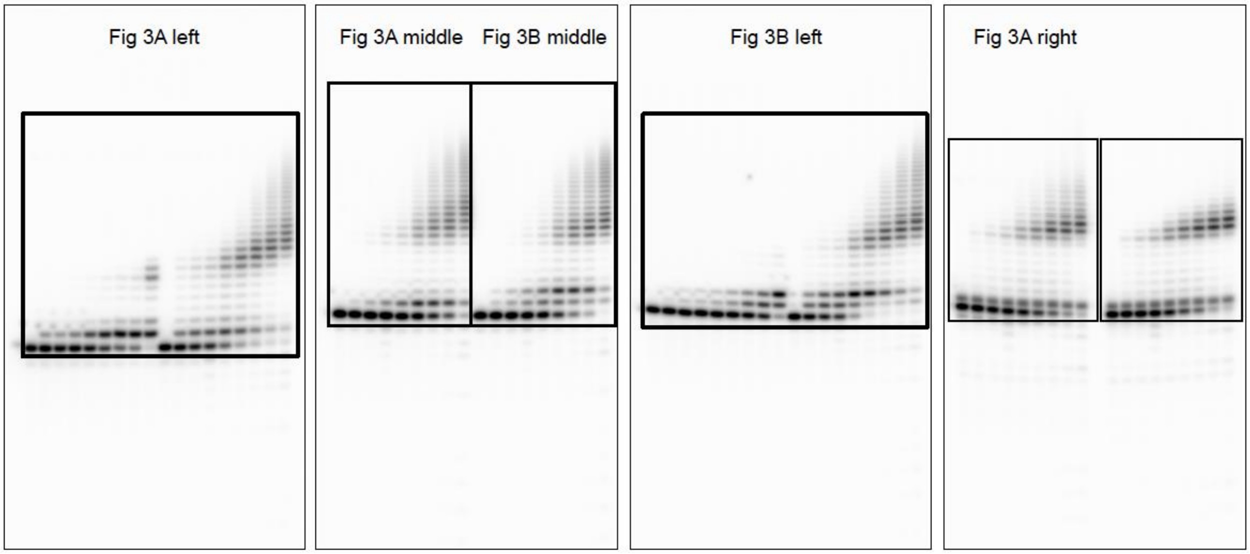
Fig 3A left
Fig 3A middle
Fig 3B middle
Fig 3B left
Fig 3A right

Fig 3C left

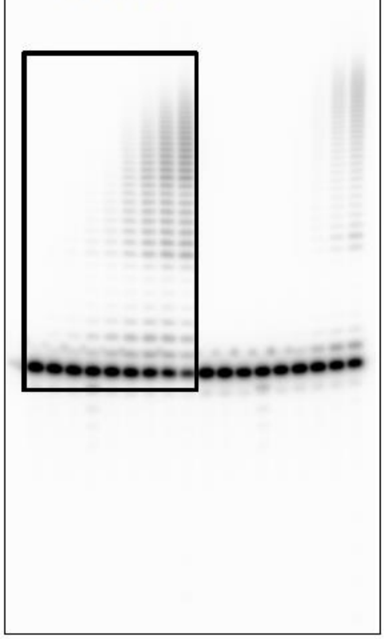

Fig 3C right

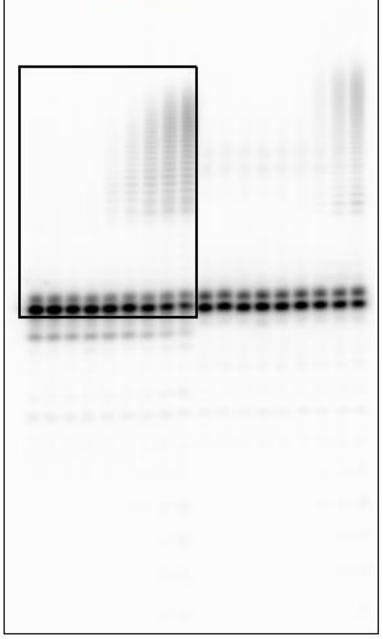

Fig 3D left Fig 3D right

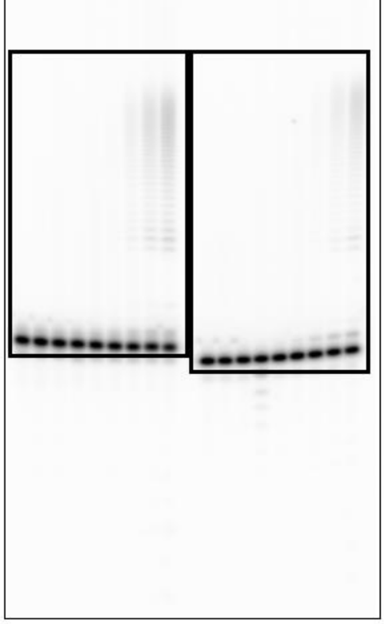

Fig 3E left Fig 3E right

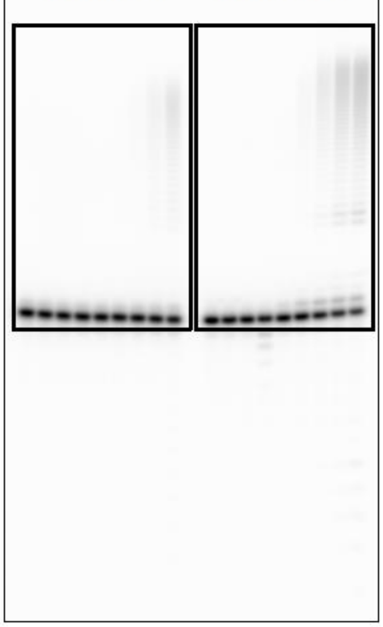

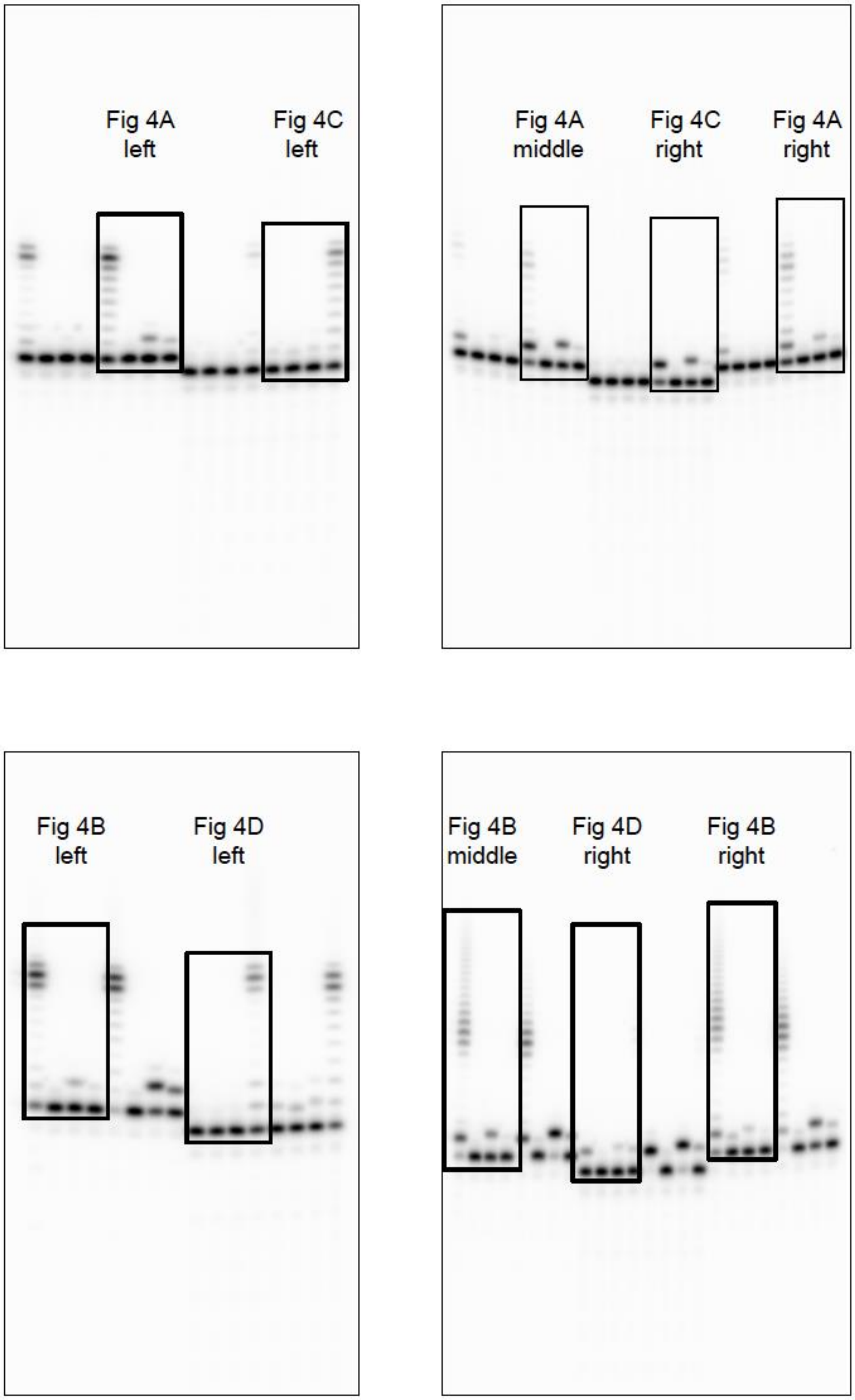


**Supplementary Figure S10.** Original gel images are shown in Figures 2, 3, and 4.

# Supplementary Table S1

**Primers and templates used for DNA polymerase assay**

| Primer/ Template | Primer | Template | Primer/Template |
|---|---|---|---|
| P/T | 5′-TTTTTTTTAGACTC | 5′-TTTTTTTTGAGTCT | 5′-TTTTTTTTAGACTC<br>TCTGAGTTTTTTTT-5′ |
| P_AP1/T | 5′-TTTTTTTTAGACT^ | 5′-TTTTTTTTGAGTCT | 5′-TTTTTTTTAGACT^<br>TCTGAGTTTTTTTT-5′ |
| P_AP3/T | 5′-TTTTTTTTAGA^TC | 5′-TTTTTTTTGAGTCT | 5′-TTTTTTTTAGA^TC<br>TCTGAGTTTTTTTT-5′ |
| P_AP5/T | 5′-TTTTTTTTA^ACTC | 5′-TTTTTTTTGAGTCT | 5′-TTTTTTTTA^ACTC<br>TCTGAGTTTTTTTT-5′ |
| P/T_AP7 | 5′-TTTTTTTTAGACTC | 5′-TTTTTTT^GAGTCT | 5′-TTTTTTTTAGACTC<br>TCTGAG^TTTTTTT-5′ |
| P/T_AP8 | 5′-TTTTTTTTAGACTC | 5′-TTTTTT^TGAGTCT | 5′-TTTTTTTTAGACTC<br>TCTGAGT^TTTTTT-5′ |
| P/T_full | 5′-TTTTTTTTAGACTC | 5′-TTTTTTTTGAGTCTAAAAAAAA | 5′-TTTTTTTTAGACTC<br>AAAAAAAATCTGAGTTTTTTTT-5′ |
| P/T_AP7_full | 5′-TTTTTTTTAGACTC | 5′-TTTTTTT^GAGTCTAAAAAAAA | 5′-TTTTTTTTAGACTC<br>AAAAAAAATCTGAG^TTTTTTT-5′ |
| P/T_AP8_full | 5′-TTTTTTTTAGACTC | 5′-TTTTTT^TGAGTCTAAAAAAAA | 5′-TTTTTTTTAGACTC<br>AAAAAAAATCTGAGT^TTTTTT-5′ |
| P/T_Tg1 | 5′-TTTTTTTTAGACTC | 5′-TTTTTTTTGAGTC**X** | 5′-TTTTTTTTAGACTC<br>**X**CTGAGTTTTTTTT-5′ |
| P/T_Tg3 | 5′-TTTTTTTTAGACTC | 5′-TTTTTTTTGAG**X**CT | 5′-TTTTTTTTAGACTC<br>TC**X**GAGTTTTTTTT-5′ |
| P/T_Tg7 | 5′-TTTTTTTTAGACTC | 5′-TTTTTTT**X**GAGTCT | 5′-TTTTTTTTAGACTC<br>TCTGAG**X**TTTTTTT-5′ |
| P/T_Tg8 | 5′-TTTTTTTTAGACTC | 5′-TTTTTT**X**TGAGTCT | 5′-TTTTTTTTAGACTC<br>TCTGAGT**X**TTTTTT-5′ |
| P_Tg2/T | 5′-TTTTTTTTAGAC**X**C | 5′-TTTTTTTTGAGTCT | 5′-TTTTTTTTAGAC**X**C<br>TCTGAGTTTTTTTT-5′ |
| P/T_Tg7_full | 5′-TTTTTTTTAGACTC | 5′-TTTTTTT**X**GAGTCTAAAAAAAA | 5′-TTTTTTTTAGACTC<br>AAAAAAAATCTGAG**X**TTTTTTT-5′ |
| P/T_Tg8_full | 5′-TTTTTTTTAGACTC | 5′-TTTTTT**X**TGAGTCTAAAAAAAA | 5′-TTTTTTTTAGACTC<br>AAAAAAAATCTGAGT**X**TTTTTT-5′ |
| P_full/T | 5′-TTTTTTTTAGACTCAAAAAAAA | 5′-TTTTTTTTGAGTCT | 5′-TTTTTTTTAGACTCAAAAAAAA<br>TCTGAGTTTTTTTT-5′ |
| P_full/T_full | 5′-TTTTTTTTAGACTCAAAAAAAA | 5′-TTTTTTTTGAGTCTAAAAAAAA | 5′-TTTTTTTTAGACTCAAAAAAAA<br>AAAAAAAATCTGAGTTTTTTTT-5′ |
| PA/TA | 5′-AAAAAAAAAGACTC | 5′-AAAAAAAAGAGTCT | 5′-AAAAAAAAAGACTC<br>TCTGAGAAAAAAAA-5′ |
| PA_AP1/TA | 5′-AAAAAAAAAGACT^ | 5′-AAAAAAAAGAGTCT | 5′-AAAAAAAAAGACT^<br>TCTGAGAAAAAAAA-5′ |
| PA_AP3/TA | 5′-AAAAAAAAAGA^TC | 5′-AAAAAAAAGAGTCT | 5′-AAAAAAAAAGA^TC<br>TCTGAGAAAAAAAA-5′ |
| PA_AP5/TA | 5′-AAAAAAAAA^ACTC | 5′-AAAAAAAAGAGTCT | 5′-AAAAAAAAA^ACTC<br>TCTGAGAAAAAAAA-5′ |
| PA/TA_AP7 | 5′-AAAAAAAAAGACTC | 5′-AAAAAAA^GAGTCT | 5′-AAAAAAAAAGACTC<br>TCTGAG^AAAAAAA-5′ |
| PA/TA_AP8 | 5′-AAAAAAAAAGACTC | 5′-AAAAAA^AGAGTCT | 5′-AAAAAAAAAGACTC<br>TCTGAGA^AAAAAA-5′ |
| PA/TA_full | 5′-AAAAAAAAAGACTC | 5′-AAAAAAAAGAGTCTTTTTTTTT | 5′-AAAAAAAAAGACTC<br>TTTTTTTTTCTGAGAAAAAAAA-5′ |
| PA/TA_AP7_full | 5′-AAAAAAAAAGACTC | 5′-AAAAAAA^GAGTCTTTTTTTTT | 5′-AAAAAAAAAGACTC<br>TTTTTTTTTCTGAG^AAAAAAA-5′ |
| PA/TA_AP8_full | 5′-AAAAAAAAAGACTC | 5′-AAAAAA^AGAGTCTTTTTTTTT | 5′-AAAAAAAAAGACTC<br>TTTTTTTTTCTGAGA^AAAAAA-5′ |

Note: THF and Tg are denoted as ^ and X, respectively.

# Supplementary Table S2

**The number of micronuclei per binucleated cell and the percentage of micronuclei-containing binucleated cells**

| Cell line | IR source | Dose (Gy) | Experiment | Cell number | No. of micronuclei | No. of micronuclei-containing cells | No. of micronuclei per cell | % of cells with micronuclei |
|---|---|---|---|---|---|---|---|---|
| U2OS | * - | 0 | Technical Replicate 1 | 100 | 7 | 6 | 0.07 | 6.0 |
| U2OS | - | 0 | Technical Replicate 2 | 100 | 8 | 8 | 0.08 | 8.0 |
| U2OS | - | 0 | Technical Replicate 3 | 200 | 9 | 9 | 0.05 | 4.5 |
| U2OS | X-rays | 2 | Technical Replicate 1 | 50 | 53 | 31 | 1.06 | 62.0 |
| U2OS | X-rays | 2 | Technical Replicate 2 | 50 | 60 | 34 | 1.2 | 68.0 |
| U2OS | X-rays | 2 | Technical Replicate 3 | 100 | 103 | 60 | 1.03 | 60.0 |
| U2OS | Carbon ions | 2 | Technical Replicate 1 | 100 | 37 | 21 | 0.37 | 21.0 |
| U2OS | Carbon ions | 2 | Technical Replicate 2 | 100 | 37 | 20 | 0.37 | 20.0 |
| U2OS | Carbon ions | 2 | Technical Replicate 3 | 50 | 27 | 16 | 0.54 | 32.0 |
| F10 | - | 0 | Technical Replicate 1 | 50 | 6 | 6 | 0.12 | 12.0 |
| F10 | - | 0 | Technical Replicate 2 | 50 | 4 | 4 | 0.08 | 8.0 |
| F10 | - | 0 | Technical Replicate 3 | 100 | 10 | 9 | 0.1 | 9.0 |
| F10 | X-rays | 2 | Technical Replicate 1 | 50 | 68 | 39 | 1.36 | 78.0 |
| F10 | X-rays | 2 | Technical Replicate 2 | 50 | 78 | 38 | 1.56 | 76.0 |
| F10 | X-rays | 2 | Technical Replicate 3 | 100 | 163 | 84 | 1.63 | 84.0 |
| F10 | Carbon ions | 2 | Technical Replicate 1 | 74 | 39 | 27 | 0.53 | 36.5 |
| F10 | Carbon ions | 2 | Technical Replicate 2 | 50 | 46 | 28 | 0.92 | 56.0 |
| F10 | Carbon ions | 2 | Technical Replicate 3 | 80 | 58 | 43 | 0.73 | 53.8 |
| G6 | - | 0 | Technical Replicate 1 | 50 | 6 | 6 | 0.12 | 12.0 |
| G6 | - | 0 | Technical Replicate 2 | 50 | 8 | 7 | 0.16 | 14.0 |
| G6 | - | 0 | Technical Replicate 3 | 100 | 8 | 6 | 0.08 | 6.0 |
| G6 | X-rays | 2 | Technical Replicate 1 | 50 | 88 | 41 | 1.76 | 82.0 |
| G6 | X-rays | 2 | Technical Replicate 2 | 50 | 76 | 41 | 1.52 | 82.0 |
| G6 | X-rays | 2 | Technical Replicate 3 | 100 | 156 | 76 | 1.56 | 76.0 |
| G6 | Carbon ions | 2 | Technical Replicate 1 | 50 | 68 | 33 | 1.36 | 66.0 |
| G6 | Carbon ions | 2 | Technical Replicate 2 | 56 | 55 | 31 | 0.98 | 55.4 |
| G6 | Carbon ions | 2 | Technical Replicate 3 | 101 | 85 | 46 | 0.84 | 45.5 |

* '-' indicates non-irradiated control sample

**The number of cells with apoptotic nucleus**

| Cell line | IR source | Dose (Gy) | Experiment | Cell number | No. of apoptotic cells | % of apoptotic cells |
|---|---|---|---|---|---|---|
| U2OS | X-rays | 2 | Technical Replicate 1 | 100 | 4 | 4.0 |
| U2OS | X-rays | 2 | Technical Replicate 2 | 100 | 1 | 1.0 |
| U2OS | X-rays | 2 | Technical Replicate 3 | 200 | 3 | 1.5 |
| U2OS | Carbon ions | 2 | Technical Replicate 1 | 102 | 18 | 17.6 |
| U2OS | Carbon ions | 2 | Technical Replicate 2 | 100 | 15 | 15.0 |
| U2OS | Carbon ions | 2 | Technical Replicate 3 | 100 | 19 | 19.0 |
| F10 | X-rays | 2 | Technical Replicate 1 | 100 | 4 | 4.0 |
| F10 | X-rays | 2 | Technical Replicate 2 | 100 | 7 | 7.0 |
| F10 | X-rays | 2 | Technical Replicate 3 | 200 | 12 | 6.0 |
| F10 | Carbon ions | 2 | Technical Replicate 1 | 100 | 20 | 20.0 |
| F10 | Carbon ions | 2 | Technical Replicate 2 | 100 | 30 | 30.0 |
| F10 | Carbon ions | 2 | Technical Replicate 3 | 200 | 64 | 32.0 |
| G6 | X-rays | 2 | Technical Replicate 1 | 100 | 6 | 6.0 |
| G6 | X-rays | 2 | Technical Replicate 2 | 100 | 7 | 7.0 |
| G6 | X-rays | 2 | Technical Replicate 3 | 200 | 19 | 9.5 |
| G6 | Carbon ions | 2 | Technical Replicate 1 | 100 | 28 | 28.0 |
| G6 | Carbon ions | 2 | Technical Replicate 2 | 100 | 31 | 31.0 |
| G6 | Carbon ions | 2 | Technical Replicate 3 | 202 | 68 | 33.7 |

# Supplementary Table S3

| Number of chromatid breaks* in U2OS cells after 2Gy of carbon ion irradiation | | | | | | | |
|---|---|---|---|---|---|---|---|
| DR-U2OS | | | F10 | | | G6 | |
| Metaphase No. | No. of Breakage | | Metaphase No. | No. of Breakage | | Metaphase No. | No. of Breakage |
| 1 | 13 | | 1 | 20 | | 1 | 13 |
| 2 | 16 | | 2 | 21 | | 2 | 11 |
| 3 | 9 | | 3 | 23 | | 3 | 19 |
| 4 | 16 | | 4 | 18 | | 4 | 15 |
| 5 | 15 | | 5 | 19 | | 5 | 16 |
| 6 | 7 | | 6 | 30 | | 6 | 19 |
| 7 | 16 | | 7 | 16 | | 7 | 21 |
| 8 | 14 | | 8 | 25 | | 8 | 24 |
| 9 | 11 | | 9 | 23 | | 9 | 13 |
| 10 | 11 | | 10 | 13 | | 10 | 12 |
| 11 | 11 | | 11 | 21 | | 11 | 28 |
| 12 | 12 | | 12 | 21 | | 12 | 31 |
| 13 | 13 | | 13 | 21 | | 13 | 20 |
| 14 | 23 | | 14 | 15 | | 14 | 29 |
| 15 | 20 | | 15 | 15 | | 15 | 28 |
| 16 | 8 | | 16 | 16 | | 16 | 19 |
| 17 | 12 | | 17 | 22 | | 17 | 36 |
| 18 | 9 | | 18 | 22 | | 18 | 27 |
| 19 | 10 | | 19 | 24 | | 19 | 30 |
| 20 | 21 | | 20 | 20 | | 20 | 24 |
| 21 | 7 | | 21 | 11 | | 21 | 25 |
| 22 | 19 | | 22 | 10 | | 22 | 20 |
| 23 | 8 | | 23 | 22 | | 23 | 21 |
| 24 | 7 | | 24 | 19 | | 24 | 35 |
| 25 | 13 | | 25 | 26 | | 25 | 17 |
| 26 | 11 | | 26 | 26 | | 26 | 20 |
| 27 | 8 | | 27 | 22 | | 27 | 22 |
| 28 | 9 | | 28 | 25 | | 28 | 30 |
| 29 | 20 | | 29 | 28 | | 29 | 14 |
| 30 | 16 | | 30 | 21 | | 30 | 20 |
| 31 | 7 | | 31 | 35 | | 31 | 15 |
| 32 | 14 | | 32 | 22 | | 32 | 24 |
| 33 | 15 | | 33 | 25 | | 33 | 26 |
| 34 | 14 | | 34 | 34 | | 34 | 24 |
| 35 | 14 | | 35 | 29 | | 35 | 32 |
| **Average** | **12.8** | | **Average** | **21.7** | | **Average** | **22.3** |
| **Fold Change** | **1** | | **Fold Change** | **1.6953125** | | **Fold Change** | **1.7421875** |

* The number of chromatid breaks was counted in each metaphase cell

## Number of chromatid breaks* in U2OS cells after 2Gy of x-ray irradiation

| DR-U2OS | | | F10 | | | G6 | |
|---|---|---|---|---|---|---|---|
| Metaphase No. | No. of Breakage | | Metaphase No. | No. of Breakage | | Metaphase No. | No. of Breakage |
| 1 | 12 | | 1 | 10 | | 1 | 10 |
| 2 | 5 | | 2 | 11 | | 2 | 8 |
| 3 | 7 | | 3 | 9 | | 3 | 5 |
| 4 | 7 | | 4 | 7 | | 4 | 12 |
| 5 | 6 | | 5 | 10 | | 5 | 8 |
| 6 | 8 | | 6 | 2 | | 6 | 9 |
| 7 | 9 | | 7 | 8 | | 7 | 10 |
| 8 | 8 | | 8 | 7 | | 8 | 3 |
| 9 | 4 | | 9 | 16 | | 9 | 8 |
| 10 | 4 | | 10 | 14 | | 10 | 20 |
| 11 | 5 | | 11 | 17 | | 11 | 11 |
| 12 | 6 | | 12 | 9 | | 12 | 8 |
| 13 | 8 | | 13 | 6 | | 13 | 10 |
| 14 | 2 | | 14 | 13 | | 14 | 17 |
| 15 | 3 | | 15 | 14 | | 15 | 10 |
| 16 | 3 | | 16 | 3 | | 16 | 10 |
| 17 | 2 | | 17 | 10 | | 17 | 12 |
| 18 | 3 | | 18 | 5 | | 18 | 6 |
| 19 | 3 | | 19 | 3 | | 19 | 9 |
| 20 | 2 | | 20 | 8 | | 20 | 13 |
| 21 | 4 | | 21 | 16 | | 21 | 6 |
| 22 | 10 | | 22 | 7 | | 22 | 9 |
| 23 | 9 | | 23 | 7 | | 23 | 8 |
| 24 | 4 | | 24 | 9 | | 24 | 7 |
| 25 | 3 | | 25 | 7 | | 25 | 14 |
| 26 | 4 | | 26 | 9 | | 26 | 16 |
| 27 | 5 | | 27 | 2 | | 27 | 9 |
| 28 | 1 | | 28 | 8 | | 28 | 12 |
| 29 | 5 | | 29 | 16 | | 29 | 3 |
| 30 | 4 | | 30 | 10 | | 30 | 7 |
| 31 | 5 | | 31 | 8 | | 31 | 4 |
| 32 | 2 | | 32 | 7 | | 32 | 6 |
| 33 | 4 | | 33 | 9 | | 33 | 8 |
| | | | | | | | |
| | | | | | | | |
| **Average** | **5.060606061** | | **Average** | **9** | | **Average** | **9.333333333** |
| **Fold Change** | **1** | | **Fold Change** | **1.778443114** | | **Fold Change** | **1.844311377** |

* The number of chromatid breaks was counted in each metaphase cell

## Number of chromatid breaks* in non-irradiated U2OS cells

| DR-U2OS | | | F10 | | | G6 | |
|---|---|---|---|---|---|---|---|
| Metaphase No. | No. of Breakage | | Metaphase No. | No. of Breakage | | Metaphase No. | No. of Breakage |
| 1 | 0 | | 1 | 4 | | 1 | 0 |
| 2 | 1 | | 2 | 3 | | 2 | 3 |
| 3 | 1 | | 3 | 0 | | 3 | 1 |
| 4 | 1 | | 4 | 1 | | 4 | 2 |
| 5 | 1 | | 5 | 3 | | 5 | 1 |
| 6 | 1 | | 6 | 2 | | 6 | 2 |
| 7 | 1 | | 7 | 0 | | 7 | 2 |
| 8 | 1 | | 8 | 1 | | 8 | 0 |
| 9 | 0 | | 9 | 0 | | 9 | 2 |
| 10 | 0 | | 10 | 1 | | 10 | 2 |
| 11 | 1 | | 11 | 1 | | 11 | 3 |
| 12 | 0 | | 12 | 1 | | 12 | 2 |
| 13 | 1 | | 13 | 2 | | 13 | 1 |
| 14 | 2 | | 14 | 1 | | 14 | 1 |
| 15 | 1 | | 15 | 0 | | 15 | 1 |
| 16 | 1 | | 16 | 2 | | 16 | 1 |
| 17 | 1 | | 17 | 1 | | 17 | 0 |
| 18 | 1 | | 18 | 0 | | 18 | 2 |
| 19 | 2 | | 19 | 0 | | 19 | 1 |
| 20 | 3 | | 20 | 0 | | 20 | 3 |
| 21 | 1 | | 21 | 2 | | 21 | 1 |
| 22 | 0 | | 22 | 2 | | 22 | 1 |
| 23 | 1 | | 23 | 3 | | 23 | 1 |
| 24 | 0 | | 24 | 0 | | 24 | 1 |
| 25 | 0 | | 25 | 2 | | 25 | 2 |
| 26 | 2 | | 26 | 2 | | 26 | 2 |
| 27 | 1 | | 27 | 1 | | 27 | 1 |
| 28 | 0 | | 28 | 2 | | 28 | 0 |
| 29 | 0 | | 29 | 1 | | 29 | 0 |
| 30 | 1 | | 30 | 0 | | 30 | 0 |
| 31 | 0 | | 31 | 1 | | 31 | 0 |
| 32 | 1 | | 32 | 1 | | 32 | 3 |
| 33 | 0 | | 33 | 2 | | 33 | 1 |
| 34 | 0 | | 34 | 0 | | 34 | 2 |
| 35 | 0 | | 35 | 4 | | 35 | 1 |
| **Average** | **0.771428571** | | **Average** | **1.314285714** | | **Average** | **1.314285714** |
| **Fold Change** | **1** | | **Fold Change** | **1.703703704** | | **Fold Change** | **1.703703704** |

* The number of chromatid breaks was counted in each metaphase cell
